# Dual polarization-enabled ultrafast bulk photovoltaic response in van der Waals heterostructures

**DOI:** 10.1038/s41467-024-49760-6

**Published:** 2024-06-25

**Authors:** Zhouxiaosong Zeng, Zhiqiang Tian, Yufan Wang, Cuihuan Ge, Fabian Strauß, Kai Braun, Patrick Michel, Lanyu Huang, Guixian Liu, Dong Li, Marcus Scheele, Mingxing Chen, Anlian Pan, Xiao Wang

**Affiliations:** 1 Key Laboratory for Micro-Nano Physics and Technology of Hunan Province, College of Materials Science and Engineering, Hunan University, Changsha, 410082 China; 2 School of Physics and Electronics, Hunan University, Changsha, 410082 China; 3 Institute of Physical and Theoretical Chemistry and LISA, University of Tübingen, Auf der Morgenstelle 18, 72076 Tübingen, Germany; 4 Key Laboratory for Matter Microstructure and Function of Hunan Province, Key Laboratory of Low-Dimensional Quantum Structures and Quantum Control of Ministry of Education, Synergetic Innovation Center for Quantum Effects and Applications (SICQEA), School of Physics and Electronics, Hunan Normal University, Changsha, 410081 China; 5 State Key Laboratory of Powder Metallurgy, Central South University, Changsha, 410083 China

**Keywords:** Two-dimensional materials, Electronic devices

## Abstract

The bulk photovoltaic effect (BPVE) originating from spontaneous charge polarizations can reach high conversion efficiency exceeding the Shockley-Queisser limit. Emerging van der Waals (vdW) heterostructures provide the ideal platform for BPVE due to interfacial interactions naturally breaking the crystal symmetries of the individual constituents and thus inducing charge polarizations. Here, we show an approach to obtain ultrafast BPVE by taking advantage of dual interfacial polarizations in vdW heterostructures. While the in-plane polarization gives rise to the BPVE in the overlayer, the charge carrier transfer assisted by the out-of-plane polarization further accelerates the interlayer electronic transport and enhances the BPVE. We illustrate the concept in MoS_2_/black phosphorus heterostructures, where the experimentally observed intrinsic BPVE response time achieves 26 ps, orders of magnitude faster than that of conventional non-centrosymmetric materials. Moreover, the heterostructure device possesses an extrinsic response time of approximately 2.2 ns and a bulk photovoltaic coefficient of 0.6 V^−1^, which is among the highest values for vdW BPV devices reported so far. Our study thus points to an effective way of designing ultrafast BPVE for high-speed photodetection.

## Abstract (web summary)

The bulk photovoltaic effect (BPVE) has potential for the realization of high conversion efficiency optoelectronic devices. Here, the authors show that combined in-plane and out-of-plane charge polarizations in MoS_2_/black phosphorus heterostructures can enhance the BPVE and reduce the extrinsic response times down to 2.2 ns.

## Introduction

The symmetry breaking in non-centrosymmetric materials brings about intriguing physical phenomena, such as second harmonic generation^1^, ferroelectricity^2^, and multiple polarization states^3^. For photoelectric conversion, the symmetry-breaking-induced asymmetric distribution of photo-excited carriers can contribute an anomalous current under zero bias, which is known as the BPVE^4^. The BPVE was initially discovered and extensively studied in ferroelectric crystals due to their perceptible spontaneous polarization^5–7^. With the theoretically predicted high conversion efficiency free from the Shockley-Queisser limit^8^, an open circuit voltage *V*_oc_ far beyond the bandgap of active material^9^ and a greater-than-unity quantum efficiency^10^ have been demonstrated in these materials. While, in pursuit of miniaturized nanoelectronics by technological development, the increase in the effect of the depolarization field against the spontaneous polarization suppresses the BPVE in conventional ferroelectric crystals at a thin limit and hinders their further application.

In addition to conventional ferroelectric crystals, recently, preserved spontaneous polarizations have been observed in emerging vdW semiconductor materials^11–13^. With the spatial designability and constructability, vdW materials through manipulating stacking orders^14^ and angles^15^ or applying a strain^16,17^ can break their centrosymmetry and hence exhibit advances of the BPVE. Benefiting from their decreased thickness comparable to the free path length, the polar two-dimensional (2D) vdW materials and structures have shown greatly enhanced BPV current density and coefficient^18^. While due to the directional carrier transporting along the nano-size tube or the quantum well, one-dimensional vdW structures^19–21^ are reported to have high-performance BPVE. Besides, combining the different vdW materials with complementary properties, such as conductive types, to form heterostructures and engineering the low-symmetric interfaces that facilitate the charge extraction may enable a further progress of the BPVE and nano devices for a specific application. However, understanding the carrier dynamics of vdW materials in BPVE generation at the low-dimensional limit and realizing a BPV device with fast speed performance remains a challenge.

Here, we propose a symmetry-engineered structure of MoS_2_/black phosphorus (BP) with in-plane and out-of-plane polarizations that combines different photocurrent generation mechanisms. A spontaneous current along the polar direction with a high ratio of anisotropy is revealed by the scanning photocurrent imaging with sub-MoS_2_-bandgap illumination. By combining charge carrier distribution simulation and time-resolved photocurrent (TRPC) measurements, we find that the heterostructure exhibits an ultrafast response time of 26 ps for the intrinsic BPVE generation, which results from the synergistic effect of the in-plane polarization and carrier accumulation assisted by the vertical built-in electric field. In comparison, the WSe_2_/BP BPV devices without the accumulation possess an intrinsic response time of approximately 1 ns, which is fifty times slower than that for the MoS_2_/BP devices. The synergistic effect in our MoS_2_/BP heterostructure further induces a 2.2 ns extrinsic response time and one of the highest BPVE coefficients (up to 0.6 V^−1^) among the existing vdW BPV devices. The large BPV coefficient, fast response speed, and wide detection range make our MoS_2_/BP BPV device promising for the next generation of the self-powered ultrathin photodetector with high efficiency.

## Results

### Design of MoS_2_/BP heterostructure with dual polarization

**Fig. 1:**
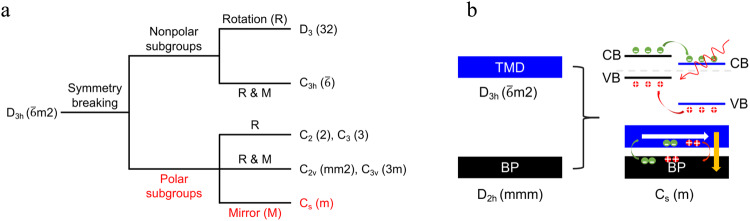
Dual polarization in transition metal dichalcogenides/black phosphorus (TMD/BP) heterostructure. **a** Symmetry breaking in TMD monolayers. Five polar groups can be obtained by breaking the *D*_3h_ symmetry for the bulk photovoltaic effect (BPVE). **b** Polar symmetry *C*_s_ obtained by stacking a TMD monolayer onto a BP monolayer. The generated in-plane and out-of-plane polarizations are illustrated by the white and the yellow arrows, respectively. The green and red arrows indicate the charge carrier transfer in conductive band (CB) and valance band (VB), respectively.

The observation of BPVE relies on the broken inversion symmetry, for which polar groups can be introduced. Figure 1a shows that breaking the *D*_3h_ symmetry of the transition metal dichalcogenides (TMD) monolayers can give rise to five types of polar groups, among them *C*_2_, *C*_2v_, and *C*_s_ can induce in-plane polarization. Besides strain engineering^22^ and effective dimensionality reduction^23^, such broken symmetries can be easily achieved by building heterostructures. For instance, the BP monolayer has a distinctly different lattice and symmetry (*D*_2h_) from the TMD monolayers. As a result, interfacing a TMD monolayer with a BP monolayer to form a TMD/BP heterostructure can lead to a symmetry reduction for the TMD monolayer from the *D*_3h_ symmetry to *C*_s_ with merely the mirror symmetry left. On the other hand, the performance of the BPVE is associated with the extraction of the charge carriers. In this regard, the built-in electric field together with the band alignment of the two constituents plays a key role in the transport and kinetics of photogenerated carriers. The ideal situation is that the built-in electric field separates the photogenerated carriers in both the overlayer and the substrate, and yields an accumulation of the carriers in each constituent. For TMD/BP heterostructure, the combination of the type-II band alignment and the build-in electric field pointing from the TMD layer to BP would favor this process (Fig. 1b). Based on the above theoretic analyses, we propose a MoS_2_/BP heterostructure with dual interfacial polarizations (other TMD/BP heterostructures see Supplementary Note 1).

**Fig. 2:**
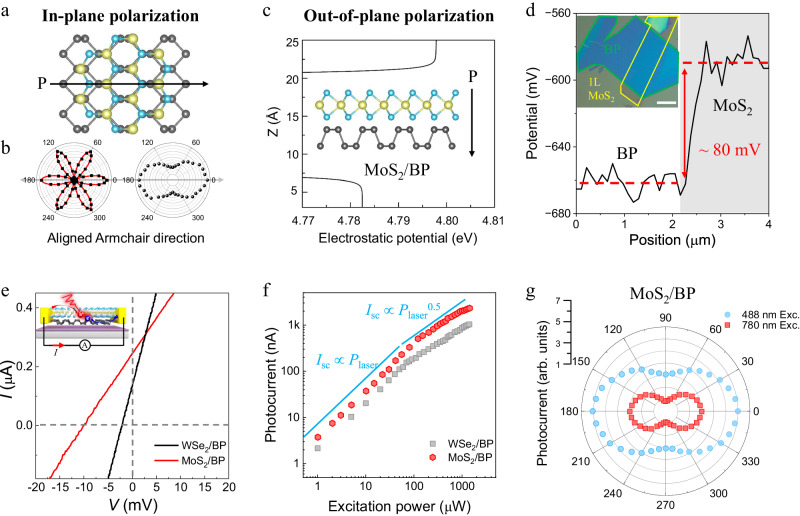
Design and BPVE of MoS_2_/BP heterostructure with in-plane and out-of-plane polarizations. **a** Schematic illustration of the in-plane polarization generation (indicated by the black arrow) in the armchair direction aligned MoS_2_/BP heterostructure. **b** Second harmonic generation (SHG) polarization in monolayer MoS_2_ (left panel) and Raman intensity ratio of the BP peak *A*_g_^2^/*A*_g_^1^(right panel), where the polar direction indicated by the gray arrow implies the determination of the armchair direction in two different materials respectively. **c** Planar averaged electrostatic potential simulation along the z direction in the armchair direction aligned MoS_2_/BP heterostructure implying a generation of the out-of-plane polarization (indicated by the black arrow). The inset shows the schematical side view of monolayer MoS_2_/BP heterostructure. **d** Kelvin probe force microscope (KPFM) line scan profile of MoS_2_/BP heterostructure showing the relative surface potential difference. The inset shows the optical image of the measured sample with an aligned armchair direction, where the green and yellow solid lines highlight the position of MoS_2_ monolayer and BP. The scale bar is 8 μm. **e** Comparison of the photocurrent in WSe_2_/BP (black) and MoS_2_/BP (red) devices under the 633 nm continuous wave (CW) laser illumination with power density *P* = 71.3 mW/cm^2^. The inset shows the schematic illustration of the TMD/BP BPV device. **f** Input laser power dependence of photocurrent intensity in MoS_2_/BP (red dots) and WSe_2_/BP (gray squares) devices. The solid lines serve as guidelines for linear and square-root dependence. **g** Photocurrent anisotropy in MoS_2_/BP device with the illumination of 488 nm CW laser (light blue) and 780 nm pulse laser (red).

We prepared MoS_2_/BP heterostructures by elaborately stacking the MoS_2_ monolayer on the BP flake with aligned armchair directions (Fig. 2a), where the crystal directions were determined (Supplementary Note 2) according to the second harmonic generation (SHG) polarization in MoS_2_ monolayer (Fig. 2b left panel) and the Raman intensity ratio in BP (Fig. 2b, right panel). The BP flake and MoS_2_ monolayer in respective p-type and n-type conductive behaviors were confirmed by the transfer characteristic measurements (Supplementary Fig. 4). The planar averaged electrostatic potential simulation along the z direction (Fig. 2c) and Kelvin probe force microscope (KPFM) measurement (Fig. 2d) simultaneously determined the out-of-plane polarization strength at tens of microvolt.

We fabricated a MoS_2_/BP device with the electrodes parallel to the mirror plane of the heterostructure and investigated its photoresponse. As a comparison, an armchair direction aligned WSe_2_ monolayer/BP heterostructure device with the same configuration was also prepared, where the out-of-plane charge carrier extraction is unfavored (Supplementary Note 3)^15^. Under the illumination of a 633 nm continuous wave (CW) laser, the MoS_2_/BP device shows a short-circuit current of approximately 250 nA, doubling that of the WSe_2_/BP device (Fig. 2e). With the increase in the input laser power, both devices display a linear to sub-linear (0.5) photocurrent transition at an excitation power of approximately 50 μW (Fig. 2f), which is different from the photovoltaic (PV) effect trend originating from the p-n junction or Schottky barrier^19^. We further used a linearly polarized laser to check the polarization of the generated photocurrent. With the rotation of the input laser, the photocurrent response in the MoS_2_/BP device displays a distinct anisotropy with its maximum along the mirror plane direction, demonstrating the broken rotation symmetry of the integrated heterostructure. Interestingly, a photoresponse was obtained under the 780 nm illumination (sub-MoS_2_ monolayer bandgap excitation) and the ratio of anisotropy from it was larger than that from 488 nm excitation (above-MoS_2_ monolayer bandgap excitation) (Fig. 2g).

### BPVE generation with sub-MoS_2_ monolayer bandgap illumination

**Fig. 3:**
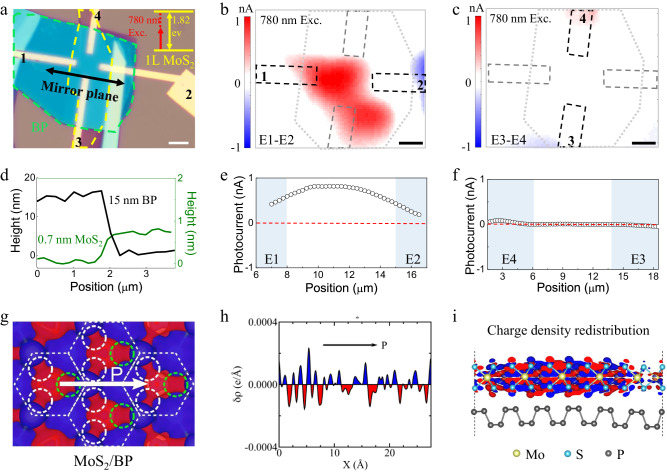
BPVE generation under sub-MoS_2_-bandgap illumination. **a** Optical image of the MoS_2_/BP BPV device with two pairs of electrodes, where the electrodes E1-E2 are parallel to the mirror plane of the device, while the E3-E4 are perpendicular to the mirror plane. The yellow and green curves outline the monolayer MoS_2_ and BP, respectively. The inset shows that the excitation with 780 nm (1.589 eV) is below MoS_2_ bandgap (1.82 eV). The white scale bar is 7 μm. **b**, **c** Scanning photocurrent microscope (SPCM) images with the electrodes E1-E2 (**b**) and E3-E4 (**c**) with the 780 nm laser. The dashed gray lines highlight the overlap region of the heterostructure. The black scale bars are 4 μm. **d** AFM line profiles of the MoS_2_/BP device indicating the thicknesses of MoS_2_ and BP being 0.7 nm and 15 nm, respectively. **e**, **f** Photocurrent line profiles along the electrodes E1-E2 (**e**) and E3-E4 (**f**) at the MoS_2_/BP heterostructure region. The shaded areas indicate the positions of electrodes. **g** Calculated polar charge density with electron accumulation (blue) and depletion (red) in MoS_2_/BP heterostructure. The dashed hexagon represents the unit cell of MoS_2_. The dashed circles show the symmetry breaking at the heterostructure interface. The white arrow denotes the charge polarization. **h** Planar averaged differential charge density in MoS_2_/BP heterostructure. **i** Side view of the in-plane polarization distribution simulation in MoS_2_/BP heterostructure, where the charge redistribution mainly occurs in MoS_2_ layer.

To determine whether the photoresponse under 780 nm (1.589 eV) illumination is derived from the BPVE, scanning photocurrent microscope (SPCM) measurements were performed without external bias. For a better demonstration of the photocurrent generation, we fabricated another MoS_2_/BP device with two pairs of crossed electrodes that were parallel (E1-E2) and perpendicular (E3-E4) to the mirror plane, respectively (Fig. 3a). The atomic force microscope (AFM) line profiles indicate that the thickness of BP was 15 nm and the thickness of MoS_2_ was 0.7 nm (Fig. 3d) for the fabricated device. With connected E1-E2 electrodes, photocurrent with unchanged polarity appeared at the entire heterostructure region and the photocurrent intensity became higher far away from the electrodes (Fig. 3b), reflecting a spontaneous charge carrier separation by the in-plane polarization. In contrast, with the connected E3-E4 electrodes, a weak photocurrent with different polarities was obtained at the near electrode regions (Fig. 3c), which was attributed to the photothermoelectric effect^24^. These phenomena could be more distinctly observed in the extracted photocurrent line profiles (Fig. 3e, f), where the photocurrent from electrodes E1-E2 was one order of magnitude larger than that of electrodes E3-E4. The reproducibility of these phenomena is provided in Supplementary Note 6. The comparison of the photocurrent pattern and intensity in different directions reflects a clear BPVE under a sub-monolayer MoS_2_ bandgap (1.82 eV) excitation^25^.

We conducted density-functional theory (DFT) calculations to analyze the generation of in-plane polarization related to this BPVE. The in-plane polarization can be illustrated by the polar charge density (Δρ), which is shown in Fig. 3g. For the freestanding MoS_2_, one can see that Δρ shows the three-fold rotation symmetry (Supplementary Fig. 9a), which is, however, absent for the MoS_2_/BP heterostructure. This asymmetry results in a net in-plane polarization, which can also be seen from the planar-averaged Δρ (Fig. 3h). One can see that there is a separation of the positive and negative charge centers. In addition, we further show that Δρ is mainly distributed at the overlayer, i.e., MoS_2_ (Fig. 3i). We calculated the in-plane polarization using the Berry phase method, which gives 0.14 pC/m for the MoS_2_/BP heterostructure (Supplementary Note 9). This value is comparable to that for bilayer γ-InSe^26^.

### Ultrafast intrinsic response and dynamic analyses of BPVE

**Fig. 4:**
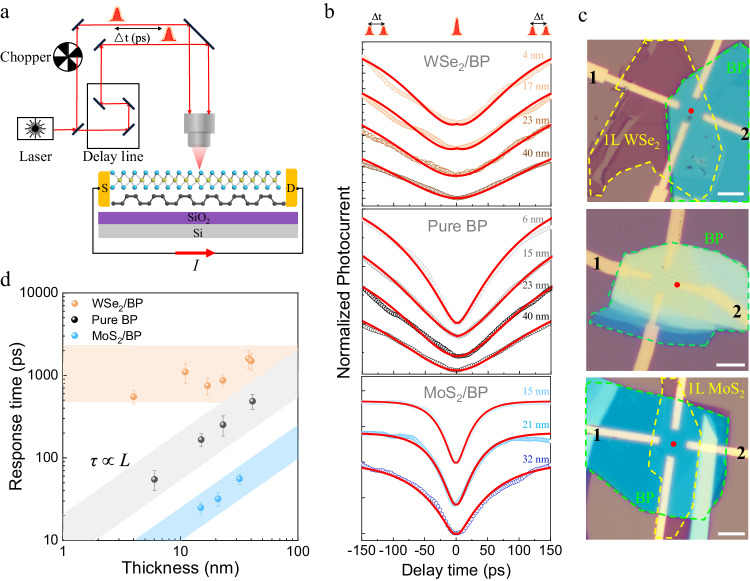
Intrinsic photocurrent response in BPV devices via time-resolved photocurrent (TRPC) technique. **a** Schematic illustration of the TRPC measurement. The Δt indicates the delay time between pump and probe beams. **b** TRPC measurements of WSe_2_/BP BPV devices, pure BP two-terminal PV devices, and MoS_2_/BP BPV devices with different BP thicknesses. The hollow circles are experimental data and the solid red lines show the fitting of the data. **c** Representative optical images in different structures showing the signal collection positions at red dots. The green and yellow dash lines indicate the position of BP and monolayer TMDs. All scale bars are 6 μm. **d** Comparison of the BP thickness (*L*) dependence of the intrinsic response time (*τ*) in WSe_2_/BP BPV devices (orange dots), pure BP two-terminal PV devices (black dots), and MoS_2_/BP BPV devices (light blue dots). The orange, gray and light blue areas serve as guides for different BP thickness dependence of the intrinsic response time. Error bars correspond to the standard deviation from multiple measurements.

To further elucidate the origin of the BPVE, we performed detailed intrinsic current dynamic measurements in the MoS_2_/BP heterostructure with the time-resolved photocurrent (TRPC) technique (Fig. 4a). For a better understanding and comparison of the photocurrent generation mechanisms, WSe_2_/BP BPV devices and pure BP two-terminal PV devices with different BP thicknesses were measured correspondingly. The spontaneous photocurrent derived from BPVE in MoS_2_/BP and WSe_2_/BP devices was collected at the center of the heterostructures while the spontaneous photocurrent derived from Schottky barrier in pure BP two-terminal devices was collected at the edge of electrodes (red dots in Fig. 4c). All intrinsic response time measurements were conducted at zero external bias. For BP two terminal devices, the intrinsic response time extended linearly from 68 ps to 488 ps with the increase in BP thickness from 4 nm to 40 nm (middle panel in Fig. 4b and black dots in Fig. 4d). We attribute this phenomenon to a defect-related recombination process^27,28^, where the photo-generated charge carriers in the middle of the thicker BP are captured from the defects with a longer recombination center than that of the surface. Meanwhile, these carriers would experience a longer out-of-plane distance to drift to the electrodes on the top. In contrast to the prominently extended response time in pure BP, the BPVE (Supplementary Note 10) dynamics from WSe_2_/BP heterostructures demonstrated intrinsic response times of approximately 1 ns (upper panel in Fig. 4b and orange dots in Fig. 4d), which are not sensitive to the thickness of BP. This phenomenon can be understood in that the generation of the photocurrent is due to a broken-symmetry-induced interfacial behavior as the in-plane polarization only distributes within a few atomic layers from the heterointerface^15^. After excitation, the charge carriers from the interface are separated by the in-plane polarization and form a BPVE. The slightly increased response time for over 40 nm WSe_2_/BP heterostructures could be attributed to the influence of the increased amount of generated charge carriers in the bottom BP, which may affect the dynamics of carriers originating from the BPVE. The two distinct photocurrent dynamic results in WSe_2_/BP and BP in turn demonstrate the different origins between the BPVE and the PV effect in respective structures. Intriguingly, regarding MoS_2_/BP devices, we observed the fastest 26 ps intrinsic response time (lower panel in Fig. 4b), much shorter than that in WSe_2_/BP heterostructures. In addition, these intrinsic response times display a linear dependence on the BP thickness (light blue dots in Fig. 4d), similar to that of the PV effect in pure BP devices. These observed phenomena in MoS_2_/BP are consistent with our proposed carrier extraction design (Fig. 1b). With the type-II band alignment and vertical p-n junction in the heterostructures, the electrons generated from the BP layer by the illumination are rapidly transferred into the monolayer MoS_2_ under the out-of-plane polarization. After that, the formed in-plane polarization can further accelerate these electrons via the BPVE. Because the electrodes parallel to the mirror plane also contact the bottom BP flake in our structures, the remaining holes in the bottom BP are collected by the electrodes and the carrier circulation is realized. In this way, the BPVE response for MoS_2_/BP heterostructure is related to the BP thickness, and its ultrafast speed is attributed to the charge carrier transfer.

Regarding the mechanism of spontaneous photocurrent, shift current is one of the main originations for BPVE. The generation of shift current in bulk non-centrosymmetric materials and TMD has been investigated by the calculation^29^ and THz emission spectroscopy^30–32^, where its typical response time was roughly 0.1 ps. However, in the shift current mechanism, displacement of electrons occurs during the optical transition process, and it does not have a contribution to the BPVE after the optical transition. Besides electron shift in real space, the BPVE is also related to the unbalanced velocity distribution in momentum space, which is referred to ballistic current. Normally, under linearly polarized light excitation, the ballistic current can occur in non-centrosymmetric non-magnetic materials, if an additional scattering process is considered^33^. For our MoS_2_/BP heterostructures, the BPVE is not only contributed by the photo-excited charge carriers at the hetero-interface but also related to the charge carriers transferred from the bottom BP, where the charge carrier transfer process may introduce electron-phonon or electron-defect interactions and contribute ballistic current.

### High-frequency extrinsic photoresponse

**Fig. 5:**
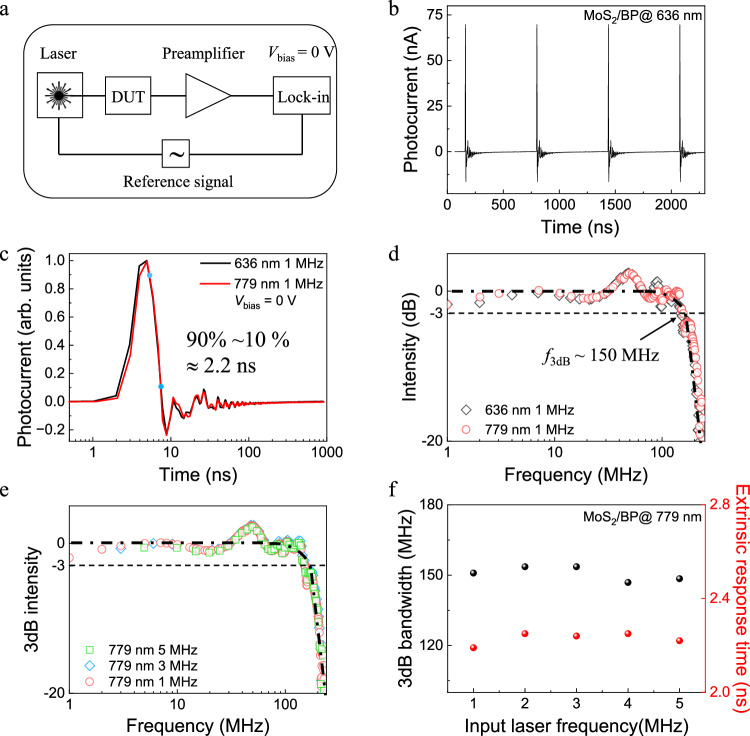
Extrinsic photocurrent response in MoS_2_/BP BPV device. **a** Experimental configuration for the extrinsic response time measurements. DUT device under test. **b** Photoresponse towards 636 nm pulse sequence under zero external bias. **c** Normalized extrinsic response time results in semi-log scale axis towards 636 nm (black) and 779 nm (red) impulse laser. The extrinsic response time defined by the 90% to 10% photocurrent decay (indicated blue dots) are approximately 2.2 ns for two different lasers. **d** Fourier transformed 3 dB bandwidth corresponding to the results in (**c**). **e** Fourier transformed 3 dB bandwidth results towards 779 nm impulse laser for different repetition frequencies. **f** Input 779 nm laser frequency dependence of extrinsic response time and the 3 dB bandwidth.

In contrast to the intrinsic response measurement which provides the upper limit speed of the device’s photoresponse, the extrinsic response investigation gives the conventional device speed and reflects the ability of a photodetector to process high-frequency signals. To this end, we determined the extrinsic response time in our MoS_2_/BP device using the experimental setup illustrated in Fig. 5a (details see Methods and Supplementary Notes 14 and 15). Upon the 636 nm pulse illumination, the device displays a constant response for several cycles as shown in Fig. 5b. We exemplarily extract a single pulse response and resolve it on a semi-log scale axis. The 90% to 10% photocurrent decay indicates an extrinsic response time of 2.2 ns (Fig. 5c, black curve). Fast Fourier transformation (FFT) was used to calculate the electrical bandwidth under the single excitation frequency and a 3 dB bandwidth of approximately 150 MHz was obtained (Fig. 5d, gray squares), which is comparable to that of state-of-art self-powered photodetectors^34,35^. We further changed the input laser to 779 nm and conducted laser repetition frequency dependent measurements. The results show a comparable response time (Fig. 5c, red curve) and 3 dB bandwidth (Fig. 5d, red dots) for all laser frequencies (Fig. 5e, f). These results further illustrate that the unique BPVE generation in our MoS_2_/BP heterostructure device can be used to detect near-infrared signals.

### Overview of BPV coefficient and response time

**Fig. 6:**
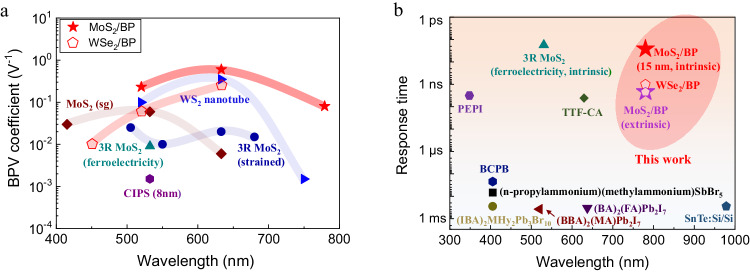
BPV coefficients and response time in various symmetry breaking devices. **a** Comparison of the BPV coefficient in various vdW BPV devices. Data for other materials are taken from the literature (WS_2_ nanotube, ref. ^19^; MoS_2_ (strain gradient), ref. ^3, 16^. R MoS_2_ (ferroelectricity), ref. ^3, 14^. R MoS_2_ (strained), ref. ^17^; CuInP_2_S_6_ (CIPS) (8 nm), ref. ^18^). The calculated maximum BPV coefficient of our MoS_2_/BP heterostructure is approximately 0.6 V^−^^1^, which is among one of the highest values in various vdW BPV devices. **b** Comparison of the response time between our TMD/BP heterostructures and other conventional ferroelectric materials. Data for other materials are taken from the literature. (3R MoS_2_ (ferroelectricity), ref. ^36^; (iso-pentylammonium)_2_(ethylammonium)_2_Pb_3_I_10_ (PEPI), ref. ^40^; tetrathiafulvalene-*p*-chloranil (TTF-CA), ref. ^41^; (C_6_H_5_CH_2_NH_3_)_2_CsPb_2_Br_7_ (BCPB), ref. ^42^; (n-propylammonium)(methylammonium)SbBr_5_ ref. ^43^; (IBA)_2_MHy_2_Pb_3_Br_10_, ref. ^44^; (BA)_2_(FA)Pb_2_I_7_, ref. ^45^; SnTe:Si/Si, ref. ^46^; (BBA)_2_(MA)Pb_2_I_7_, ref. ^47^).

We now quantitatively compare the BPVE performance of our MoS_2_/BP heterostructure with other recently reported vdW materials and ferroelectric materials. In an evaluation of the BPVE strength in our MoS_2_/BP heterostructure, we calculated the BPV coefficient based on: *j*_*i*_ = *β*_*ilm*_*E*_*l*_*E*_m_^*^I, where *j*_*i*_ is the current density for BPVE, *β*_*ilm*_ is the BPV coefficient, *E*_*l*_ and *E*_m_^*^ are the light polarization unit vectors and *I* is the light intensity. Here we used the 633 nm, 520 nm, and 779 nm CW laser with the excitation power of 71.3 mW/cm^2^. For the MoS_2_/BP device with a channel length of 7 µm and a photosensitive area of 49 µm, the calculated BPV coefficients were 0.6 V^−^^1^, 0.23 V^−^^1^ and 0.08 V^−^^1^, respectively. Comparing the BPV coefficient with that reported in other vdW materials, for instance, 3R-MoS_2_, WS_2_ nanotube, and CuInP_2_S_6_ (CIPS) (Fig. 6a), we found that our MoS_2_/BP device demonstrates one of the highest values, indicating the improved strength by making use of dual interfacial polarizations. Besides, the short-circuit current density *j*_*i*_ is also plotted as the function of incident power density *P* (Supplementary Note 17), where our MoS_2_/BP heterostructure is also among the highest results.

In terms of speed performance, the extrinsic response time of our MoS_2_/BP (15 nm BP) device is around 2.2 ns, which is five orders of magnitude faster than that of most recently reported ferroelectric perovskite at a time scale of approximately ten to hundreds of microseconds (Fig. 6b). In addition, the intrinsic response time down to 26 ps is comparable to that in graphene-incorporated 3R MoS_2_^36^. This intrinsic response time corresponds to a photodetection bandwidth of *f* = 0.55/*τ* = 21 GHz, which almost reaches the highest reported photo-switching speed for BPV photodetectors. It should be noted that further improved response speed can be reached with decreasing the BP thickness.

In summary, we have demonstrated a high efficiency and ultrafast BPVE in polarization-engineered MoS_2_/BP van der Waals heterostructure device. With the synergic effect of in-plane and out-of-plane dual-polarization, the heterostructure displays a 0.6 V^−^^1^ BPV coefficient and an intrinsic 26 ps response time, which suggests a possibility for the enhancement of the photoresponse by combining different photocurrent generation mechanisms. Moreover, a 2.2 ns extrinsic response time and 150 MHz 3 dB bandwidth independent of the laser frequencies are obtained, demonstrating the potential of the vdW BPV device for high-frequency photoswitching applications. Our results provide a perspective of the BPV device for the ultrathin self-powered photodetectors with fast response speed.

## Methods

### Device fabrication

The pure BP devices and TMD/BP devices were both fabricated based on mechanically exfoliated materials with the all-dry transfer method. Before transfer, BP or TMD flakes were first mechanically exfoliated from single crystals (purchased from Nanjing Muke Nanotechnology Co., Ltd.) onto transparent polydimethylsiloxane (PDMS), and the crystal directions of BP flakes and TMD flakes on PDMS were determined via polarized Raman spectrum and polarized SHG, respectively. Thereafter, the BP flake was placed onto a silicon substrate with a 300 nm-thick silicon dioxide layer. The TMD flake was then aligned to the BP flake and transferred onto it with the help of a microscope. Cr/Au (10 nm/50 nm) conducting electrodes on top of 2D BP or TMD/BP with 7 μm channel length were fabricated using standard electron beam lithography (EBL), metal thermal evaporation, and lift-off processes.

### Basic characterizations

Atomic force microscopy (AFM) (Bruker Dimension Icon) in the tapping mode was used to identify the thickness of the samples. Raman measurements of the samples were taken using a confocal microscope (WITec, alpha-300) equipped with a 50× objective lens (Zeiss EC Epiplan). The excitation source of the Raman was a 488 nm continuous-wave laser, and the laser beam was focused to the size of about 1 μm on the samples. The SHG measurements of the samples in reflection configuration were performed using the same confocal microscope with 800 nm laser pulses (repetition rate of 80 MHz, pulse width of 100 fs). The electrical properties were measured with an Agilent-B1500 semiconductor analyzer in a LakeShore vacuum chamber of 10^−4^ Pa. The extrinsic response measurements were performed on a probe station using a picosecond pulse laser drive (Taiko PDL M1, PicoQuant) equipped 636 nm and 779 nm laser head with a pulse width of < 500 ps. The generated photocurrent was pre-amplified with a FEMTO HSA-Y-1-60 high-speed current amplifier, after that, it was collected with a Zurich Instruments UHFLI lock-in amplifier.

### SPCM and TRPC measurements

SPCM and TRPC were performed on our home-built setup. In SPCM measurements, a 780 nm fiber laser (NPI Rainbow 780 OEM) with a pulse width of 80 fs or a 488 nm continuous wave laser was chopped by a mechanical chopper at 1050 Hz, and then focused onto the sample by a long working distance objective (Olympus LMPLFLN 50×) near the diffraction limit. The generated photocurrent was collected by a lock-in amplifier (Stanford SR830) at the chopped frequency with a background noise of approximately 0.2 pA. The SPCM measurements with the resolution close to the diffraction limit were performed by raster scanning the entire device mounted on a piezoelectric translation stage (Piezoconcept LT3) according to the fixed laser spot. In TRPC studies, a 780 nm pulse laser was split into two beams to form a pump-probe measurement configuration, and the probe beam was chopped so that the lock-in amplifier could only measure its photocurrent. The pump beam was delayed by different path lengths, with the delay time precisely controlled by a mechanical delay stage (Thorlabs DDSM100/M). The pump and probe beams were recombined after the delay line stage, and focused onto the sample using the same objective. The temporal resolution of the TRPC set-up is approximately 1 ps. The electric field of the input laser was always parallel to the armchair direction of the BP flakes or heterostructures in all TRPC measurements to ensure photocurrent saturation.

### Intrinsic response time fitting

Global fitting of the TRPC signals was performed using the equation: $\frac{{PC}(\Delta t)}{{PC}\left(\Delta t\to {\infty}\right)}$ = 1 - *A*${e}^{\frac{-|\Delta t|}{\tau }}$, where amplitudes A and time constants τ are the fitting parameters. The exponential time constant *τ* gives the intrinsic response time of the devices.

### DFT simulation

A slab structure was used to model the BP monolayer, TMD monolayers, and their heterostructures. A vacuum distance of 20 Å was set between adjacent slabs to avoid interlayer interaction in the periodic boundary condition. The in-plane lattice constants of the BP monolayer are 4.626 Å for ***a*** and 3.299 Å for ***b***, respectively. As for WSe_2_ and MoS_2_ monolayers, the lattice constants are 3.323 Å and 3.184 Å, respectively. In the heterostructures, a 5 × 1 supercell was used for the BP monolayer, which has a small lattice mismatch (< 1%) with a 4$\sqrt{3}$ × 1 supercell of WSe_2_. For the heterostructures of BP/MoS_2_, the BP is in a 6 × 1 supercell and the MoS_2_ monolayer has a 5$\sqrt{3}$ × 1 supercell. Density-functional theory (DFT) calculations were performed using the Vienna ab initio Simulation Package (VASP)^37^. The projector augmented wave method was used to construct pseudopotentials^38^. The plane-wave energy cutoff was set to 400 eV. The exchange-correlation functional was treated using the generalized gradient approximation as parametrized by Perdew, Burke, and Ernzerhof^39^. The Brillouin zone was sampled by 12 × 12 × 1 and 2 × 12 × 1 k-meshes for freestanding TMD monolayers and the heterostructures, respectively. Structural relaxations were done with a threshold of 10^−2^ eV Å^−1^ for the residual force on each atom. In the calculations of heterostructure, vdW dispersion forces between the adsorbate and the substrate were accounted for by using the semiempirical DFT-D3 method. The polarizations were computed by using the Berry phase method. To investigate the interface effects on the charge redistribution in TMD monolayers, we define the polar charge density as Δρ = Δρ_i_ − Δρ_f_, where Δρ_i_ and Δρ_f_ represent the density difference for the interfaced and freestanding TMD monolayers, respectively. Δρ_i_ is defined as ∆ρ_i_ = ρ_i_tot_ − ρ_i_TM_ − ρ_i_X_ − ρ_BP_, where ρ_i_tot_ represents the total charge density of the TMD/BP heterostructures, ρ_i_TM_, ρ_i_X_, and ρ_BP_ denote the ones of the isolated Mo/W, S/Se, and BP monolayer in the interface structures, respectively. ∆ρ_f_ is calculated by ∆ρ_f_ = ρ_f_tot_ − ρ_f_TM_ − ρ_f_X_, where ρ_f_tot_, ρ_f_TM_, and ρ_f_X_ denote the charge densities of the freestanding TMD monolayer, isolated Mo/W and S/Se atoms in the system, respectively.

### Supplementary information

Supplementary Information

Peer Review File

## Data Availability

Relevant data supporting the key findings of this study are available within the article and the Supplementary Information file. All raw data generated during the current study are available from the corresponding authors upon request.
