# Supplementary material for: Dual polarization-enabled ultrafast bulk photovoltaic response in van der Waals heterostructures
Source: Nat Commun. 2024 Jun 25;15:5355. doi: 10.1038/s41467-024-49760-6 (PMC11199638; doi:10.1038/s41467-024-49760-6)
Supplement: Supplementary file 1 — Supplementary Information [file 41467_2024_49760_MOESM1_ESM.pdf]

Supplementary Information

Dual polarization-enabled ultrafast bulk photovoltaic response in van der Waals heterostructures

Table of Contents:

Supplementary Notes:

Note 1: Density-functional theory (DFT) simulation of electrostatic potential in TMD/BP heterostructures with in-plane polarization

Note 2: Determination of the armchair direction of black phosphorus (BP) flakes.

Note 3: Comparison of potential difference for 1L WSe_2/BP and 1L MoS_2/BP heterostructures

Note 4: Determination of the conductive types for MoS_2 monolayer and BP

Note 5: Power dependence of I-V characteristic curves for MoS_2/BP and WSe_2/BP devices

Note 6: The repeatability of the BPVE in MoS_2/BP devices with the 780 nm laser

Note 7: Planar averaged differential charge density in freestanding monolayer MoS_2 and WSe_2/BP heterostructure

Note 8: SPCM image of monolayer MoS_2 device with the 780 nm laser

Note 9: Calculation of in-plane spontaneous electronic polarization in different TMD/BP heterostructures

Note 10: BPVE generation in WSe_2/BP devices

Note 11: Supplementary results of pure BP device

Note 12: Band alignment simulation of MoS_2/BP heterostructure

Note 13: Supplementary power-dependent TRPC results for MoS_2/BP device

Note 14: Extrinsic response time of pure BP two-terminal device

Note 15: Supplementary extrinsic photoresponse of MoS_2/BP device

Note 16: Extrinsic response time and 3-dB bandwidth of WSe_2/BP device

Note 17: Comparison of short-circuit current density in various vdW BPV devices

Note 18: Discussion of the influence of BP layers on calculations and BPVE generation

Supplementary Note 1: Density-functional theory (DFT) simulation of electrostatic potential in TMD/BP heterostructures with in-plane polarization

To demonstrate the uniqueness of our MoS_2/BP heterostructure, the out-of-plane average electrostatic potentials calculations of different TMD/BP heterostructures were conducted. Towards the MoS_2/BP heterostructure, asymmetric electrostatic potential induced out-of-plane interfacial electric field points from the MoS_2 layer to the BP layer (Supplementary Fig. 1a), which promotes the transfer of electrons from BP to MoS_2 and favors the BPVE generation. However, regarding the other three heterostructures, the interfacial electric fields point from the BP layer to the TMD layers (Supplementary Fig. 1b-d). This leads to an opposite direction of the photo-generated electron collection and hence decreases the BPVE efficiency.

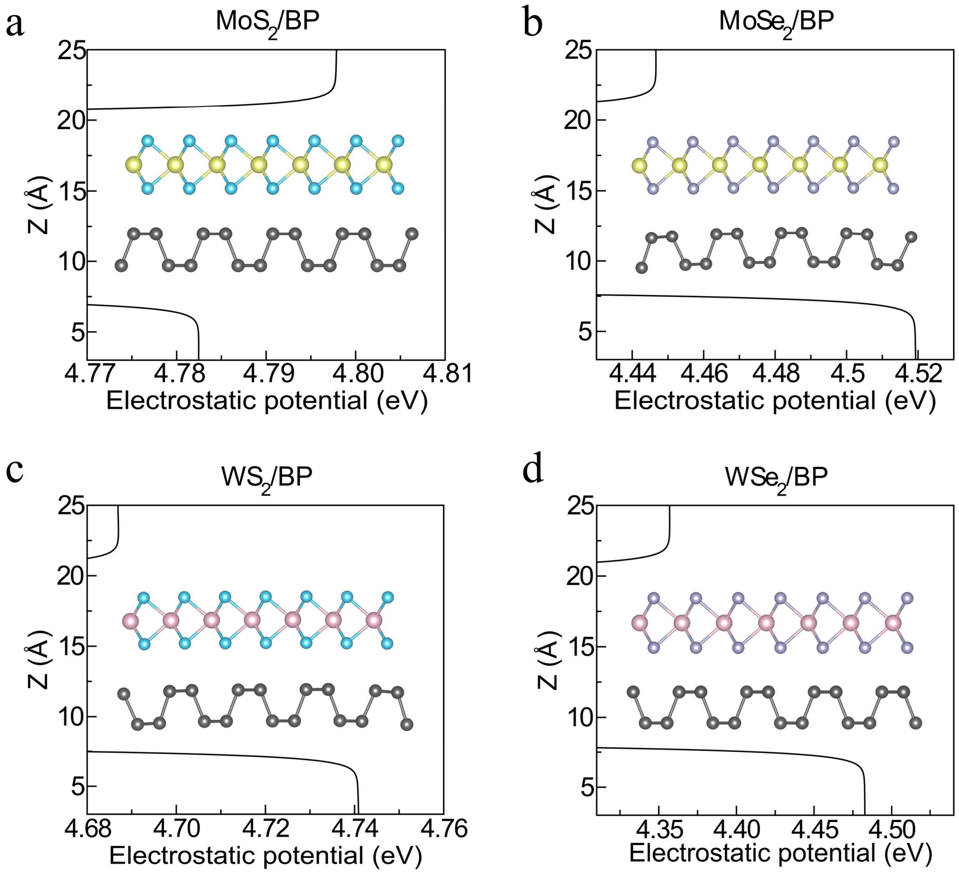

Supplementary Figure 1. Comparison of planar averaged electrostatic potentials in different TMD monolayer/BP heterostructures with in-plane polarization. a-d, Density-functional theory (DFT) simulations of electrostatic potential along z direction in MoS_2/BP (**a**), $MoSe_2$/BP (**b**), WS_2/BP (**c**), and WSe_2/BP (**d**).

Supplementary Note 2: Determination of the armchair direction of black phosphorus (BP) flakes

To prepare transition metal dichalcogenides (TMD)/BP heterostructures with aligned armchair direction, we first determined the armchair directions of individual TMD and BP flakes. The armchair direction in TMD was commonly confirmed as the petal direction (horizontal direction in the left panel of Fig. 1b in the main text) in the second harmonic generation (SHG) polarization measurement. For the determination of armchair direction in BP flakes, we performed polarization-resolved Raman spectroscopy (Supplementary Fig. 2a). When the polarization of the input laser is parallel to the BP armchair direction, the Raman intensity ratio A_g^2/A_g^1 of the BP displays a maximum value (black curve in Supplementary Fig. 2b), while when the polarization of the input laser is parallel to the BP zigzag direction, the Raman intensity ratio A_g^2/A_g^1 of the BP displays a minimum value (red curve in Supplementary Fig. 2b)[1]. The polar plot of Raman intensity ratio in the main text (Fig. 2b right panel) was conducted via the rotation of the polarization of the input laser for 360 degrees and the polar direction indicates the armchair direction of BP (horizontal direction in the right panel of Fig. 2b in the main text).

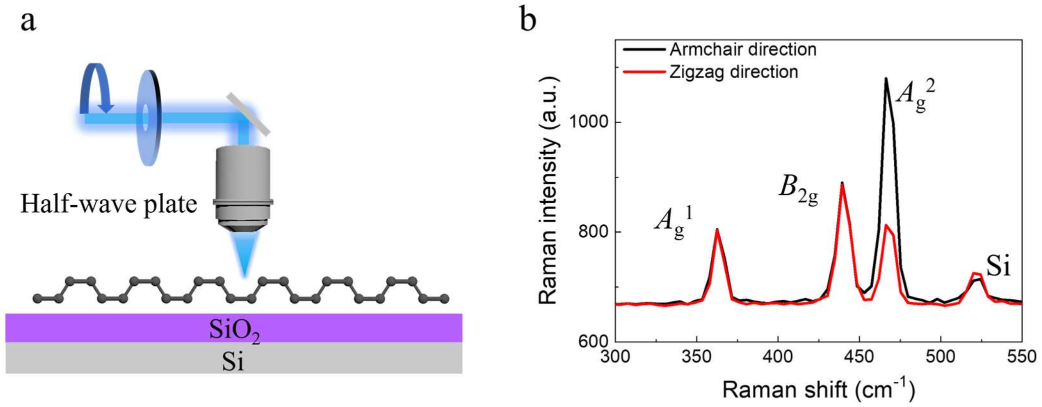

Supplementary Figure 2. Determination of the armchair direction of BP. a, Schematic of the determination of the BP's armchair direction via rotation of the polarization of the input laser. **b**, Raman spectra of BP with the input laser parallel (black) and perpendicular (red) to the armchair direction.

Supplementary Note 3: KPFM result and carrier extraction analysis for 1L WSe_2/BP heterostructure

To confirm our DFT calculation and show the uniqueness of the MoS_2/BP heterostructure, we fabricated 1L WSe_2/BP and 1L MoS_2/BP heterostructures in one sample (Supplementary Fig. 3a). In the KPFM line profile (Supplementary Fig. 3b), the potential difference between WSe_2 and BP is 30 mV, which is much smaller than that between MoS_2 and BP. The result indicates that a p-p junction is formed in the WSe_2/BP heterostructure and the out-of-plane polarization points from BP to WSe_2, which exactly corresponds to our DFT simulation (Supplementary Fig. 1c). In this case, we consider that though the formed out-of-plane polarization favors a hole transfer from BP to monolayer WSe_2, the type-I band alignment (Supplementary Fig. 3c) would suppress this effect. Hence, the out-of-plane polarization in the WSe_2/BP heterostructure would not accelerate the carrier extraction.

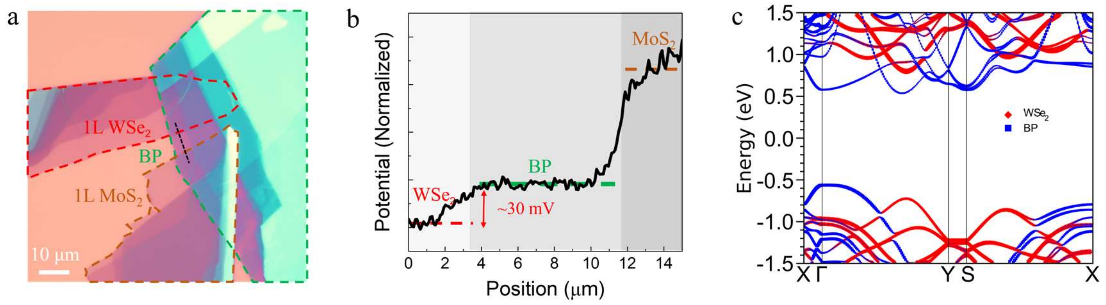

Supplementary Figure 3. **KPFM result for 1L WSe_2/BP and 1L MoS_2/BP heterostructures. a**, Optical image of 1L WSe_2/BP and 1L MoS_2/BP heterostructures in one sample. The dashed lines in red, greed and dark yellow highlight the position of 1L WSe_2, BP and 1L MoS_2, respectively. **b**, KPFM line profile showing the relative potential of the three materials. The scanned line corresponds to the black line in shown (**a**). **c**, Calculated energy band structures of the WSe_2/BP heterostructure.

Supplementary Note 4: Determination of the conductive types for MoS_2 monolayer and BP

We determined the conductive types of our MoS_2 monolayer and BP by transfer characteristic curves, where prominent p-type and n-type carrier behaviors for BP flake (Supplementary Fig. 4 left) and MoS_2 monolayer (Supplementary Fig. 4 right) were confirmed respectively.

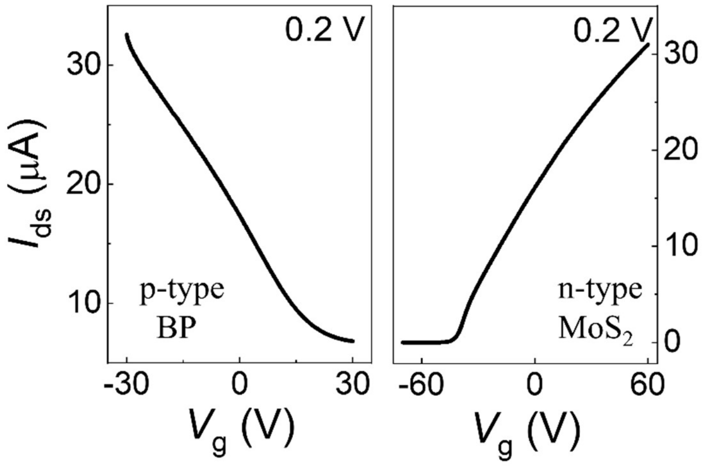

Supplementary Figure 4. Transfer characteristic curves of BP (left) and MoS_2 monolayer (right). The p-type behavior of BP and the n-type behavior of MoS_2 monolayer are confirmed by the transfer characteristics.

Supplementary Note 5: Power dependence of I-V characteristic curves for MoS_2/BP and WSe_2/BP devices

We conducted the power dependent I-V characteristic measurements for MoS_2/BP (Supplementary Fig. 5a) and WSe_2/BP (Supplementary Fig. 5b) devices with a 633 nm continuous wave (CW) laser. With the increase in laser power from 0 mW/cm^2 (dark) to 71.3 mW/cm^2, both devices demonstrate an increase in spontaneous photocurrent intensity. Compared with WSe_2/BP devices with a prominent photo-conductivity change, an almost invariant photo-conductivity reflecting as the unchanged slope in the I-V characteristic curve was obtained in the MoS_2/BP device. This phenomenon was also found in previously reported graphene-incorporated 3R-MoS_2 BPV device[2].

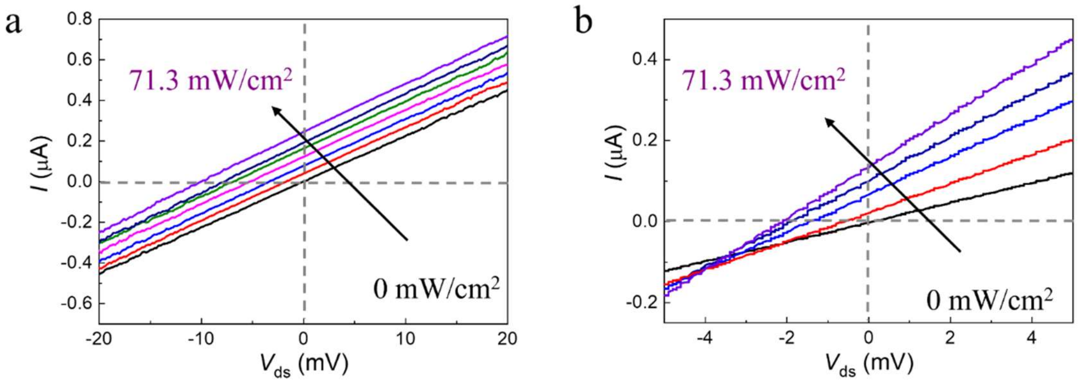

Supplementary Figure 5. Power dependence of I-V characteristic curves. a and **b,** I-V characteristic curves in MoS_2/BP (**a**) and WSe_2/BP (**b**) showing two different spontaneous photocurrent trends with the input 633 nm laser power from 0 mW/cm^2 to 71.3 mW/cm^2.

Supplementary Note 6: The reproducibility of the BPVE in other MoS_2/BP devices with the 780 nm laser

The reproducibility of the BPVE generation in different MoS_2/BP heterostructures with below monolayer MoS_2 bandgap illumination (780 nm laser) was shown in Supplementary Fig. 6-8. Towards MoS_2/BP heterostructures with different BP thicknesses (17 nm, 32 nm, and 21 nm), the spontaneous currents all appeared at the whole heterostructure region when electrodes parallel to the mirror plane were connected.

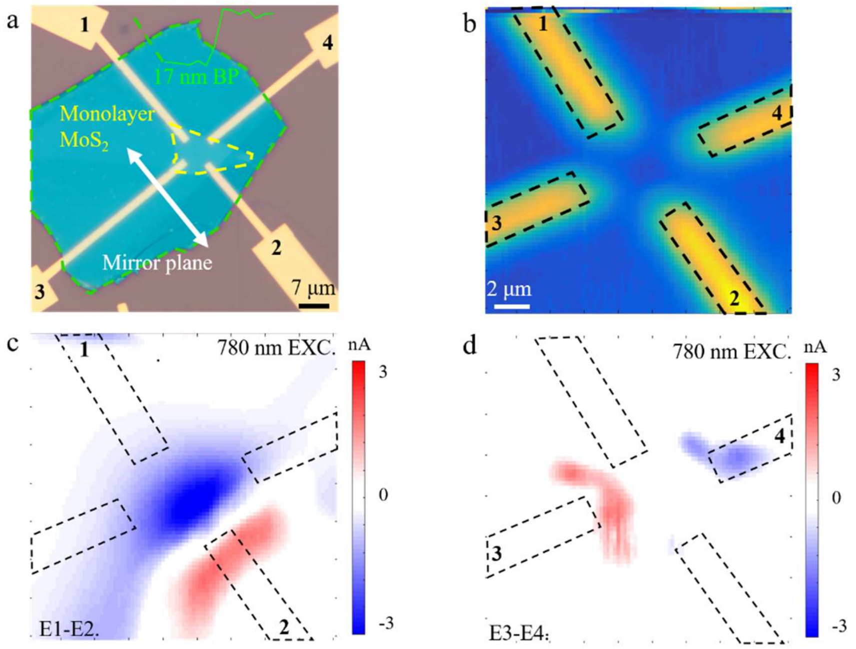

Supplementary Figure 6. BPVE generation in MoS_2/17 nm BP device with the 780 nm laser. a and **b**, Optical (**a**) and reflective (**b**) images of the MoS_2/17 nm BP BPV device with two pairs of electrodes, where the electrodes E1-E2 are parallel to the mirror plane of the device. **c**, The SPCM image with the electrodes E1-E2. **d**, The SPCM image with the electrodes E3-E4.

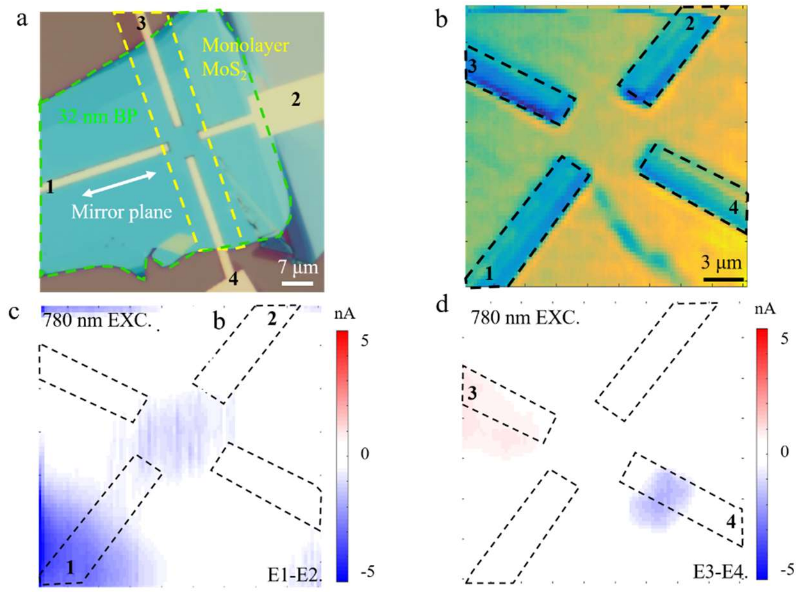

Supplementary Figure 7. BPVE generation in MoS_2/32 nm BP device with the 780 nm laser. a and **b**, Optical (**a**) and reflective (**b**) images of the MoS_2/32 nm BP BPV device with two pairs of electrodes, where the electrodes E1-E2 are parallel to the mirror plane of the device. **c**, The SPCM image with the electrodes E1-E2. **d**, The SPCM image with the electrodes E3-E4.

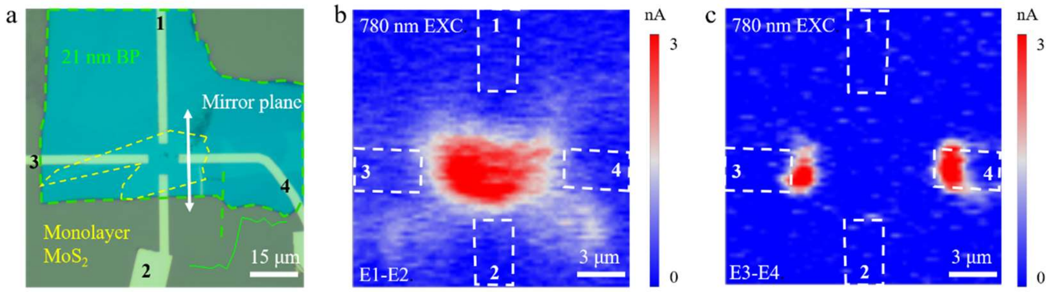

Supplementary Figure 8. BPVE generation in MoS_2/21 nm BP device with the 780 nm laser. a, Optical image of the MoS_2/21 nm BP BPV device with two pairs of electrodes, where the electrodes E1-E2 are parallel to the mirror plane of the device. **b**, The SPCM image with the electrodes E1-E2. **c**, The SPCM image with the electrodes E3-E4. Here, the absolute value of the photocurrent intensity was collected without polarity.

Supplementary Note 7: Planar averaged differential charge density in freestanding monolayer MoS_2 and WSe_2/BP heterostructure

In a comparison of MoS_2/BP heterostructure, the planar averaged charge density distribution in the freestanding monolayer MoS_2 and WSe_2/BP heterostructure were calculated correspondingly. For freestanding monolayer MoS_2, the charge density distribution displays a typical three-fold rotation symmetry, and no in-plane polarization was found (Supplementary Fig. 9a). Meanwhile, in the differential charge density calculation, a periodic result in contrast to that in heterostructures can be observed (Supplementary Fig. 9b). For WSe_2/BP heterostructure, a similar symmetry-breaking result to the MoS_2/BP heterostructure in the main text was obtained, which demonstrates that the origination of the in-plane polarization is derived from the reduced rotation symmetry by integration of hexagonal crystal structure with orthorhombic crystal structure.

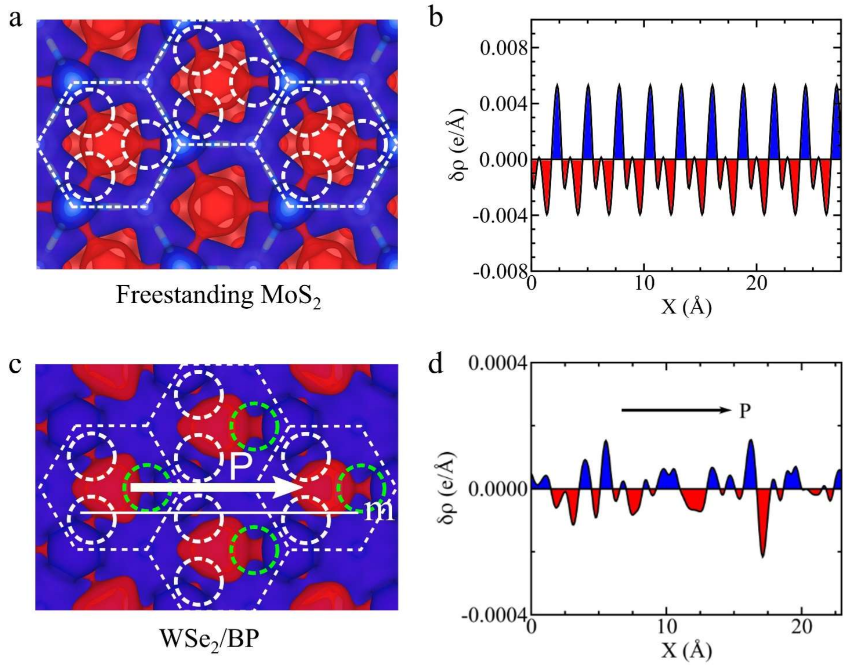

Supplementary Figure 9. Planar averaged differential charge density in freestanding monolayer MoS_2 and WSe_2/BP heterostructure. a and **c**, The planar averaged charge density distribution in freestanding monolayer MoS_2 (**a**) and WSe_2/BP heterostructure (**c**). **b** and **d**, Differential charge density in freestanding monolayer MoS_2 (**b**) and WSe_2/BP heterostructure (**d**).

Supplementary Note 8: SPCM image of monolayer MoS_2 device with the 780 nm laser

To compare the BPVE in the MoS_2/BP device, the scanning photocurrent microscope (SPCM) measurement was conducted in a pure monolayer MoS_2 device with the 780 nm laser. Here, a thin-layered h-BN flake was firstly transferred underneath the monolayer MoS_2 for its protection. The electrodes were fabricated along zigzag direction of monolayer MoS_2 (Supplementary Fig. 10a) such that the generated charge carriers can be separated via armchair direction similar to the BPVE collection configuration in MoS_2/BP device. In difference with the spontaneous current distribution concentrating on the heterostructure region far away from the electrodes, the photoresponse in monolayer MoS_2 device demonstrates a prominent photothermoelectric effect near the electrodes (Supplementary Fig. 10b and 10c) similar to previous results[3,4]. This photoresponse difference corresponds to the charge density distribution simulation results, where the in-plane polarization exists in the MoS_2/BP heterostructure rather than monolayer MoS_2.

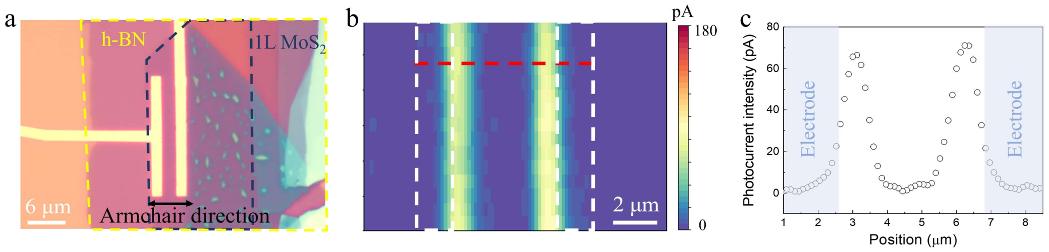

Supplementary Figure 10. Spatial photoresponse in monolayer MoS_2 device with the 780 nm laser. a, Optical image of monolayer MoS_2 device. The black arrow indicates the armchair direction of monolayer MoS_2. **b**, SPCM image of monolayer MoS_2 device. The dashed white boxes indicate the position of the electrodes. The generated photocurrent intensity value without polarity was collected. **c**, Photocurrent line profile extracted from the dashed red line in (**b**). The light blue boxes indicate the position of electrodes.

Supplementary Note 9: Calculation of in-plane spontaneous electronic polarization in different TMD/BP heterostructures

The spontaneous polarization calculations derived from the Berry phase[5] were conducted to compare the in-plane electronic polarization intensity in different TMD/BP heterostructures (Supplementary Tab. 1). Here, the comparable intensity values between WSe_2/BP and MoS_2/BP heterostructures indicate that the photocurrent density difference (Fig. 2e in the main text) is not due to the in-plane electronic polarization intensity variation but the out-of-plane polarization.

System	P_s (pC/m)
WS_2/BP	0.090
WSe_2/BP	0.139
MoS_2/BP	0.140
$MoSe_2$/BP	0.159

Supplementary Table1. The calculated spontaneous polarization Ps (pC/m per formula unit of TMD) of TMD/BP.

Supplementary Note 10: BPVE generation in WSe_2/BP devices

The SPCM results of the WSe_2/BP devices for BP thicknesses 17 nm, 4 nm, 10 nm, and 40 nm are shown in Supplementary Fig. 11-14. Here the energy (1.589 eV) of the input 780 nm laser is within the absorption edge of the monolayer WSe_2 (1.55 eV). To prevent degradation of the heterostructures with thin BP, a lower excitation power was used for the sample with 4 nm thick BP. For a typical WSe_2/BP device with 17 nm BP thickness (Supplementary Fig. 11), we extracted a photocurrent intensity from different crystal directions and presented photocurrent line profiles. The BPVE generation along the mirror plane was confirmed by the enhanced photoresponse at the heterostructure center away from electrodes.

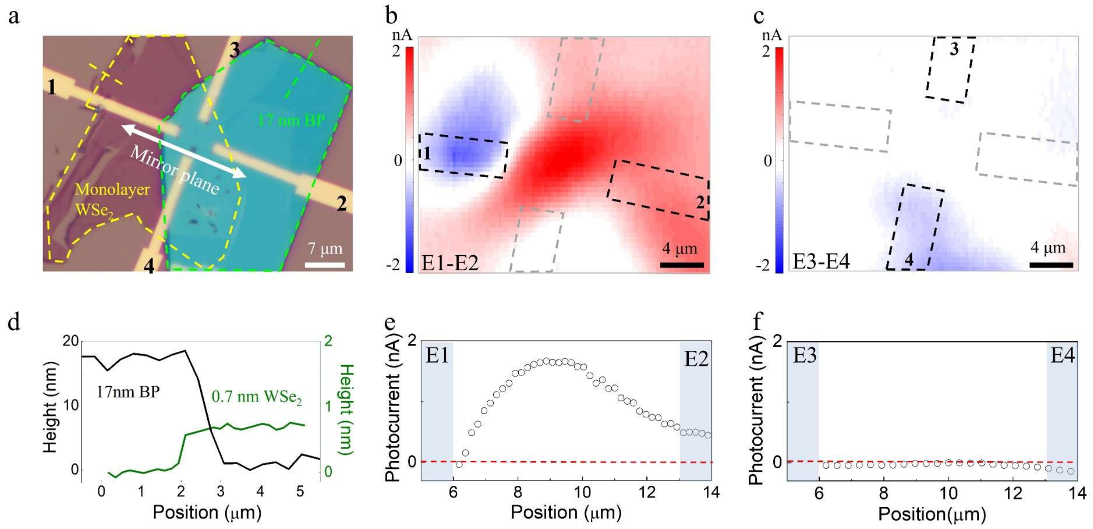

Supplementary Figure 11. BPVE generation in WSe_2/17 nm BP with the 780 nm laser. **a**, Optical image of the WSe_2/17 nm BP BPV device. **b** and **c**, SPCM image with the electrodes E1-E2 (**b**). and electrodes E3-E4 (**c**). **d**, AFM line profiles of the WSe_2/BP device indicating the thicknesses of WSe_2 and BP being 0.7 nm and 17 nm. **e** and **f**, The photocurrent line profiles along the electrodes E1-E2 (**e**) and E3-4 (**f**) at the WSe_2/BP heterostructure regions.

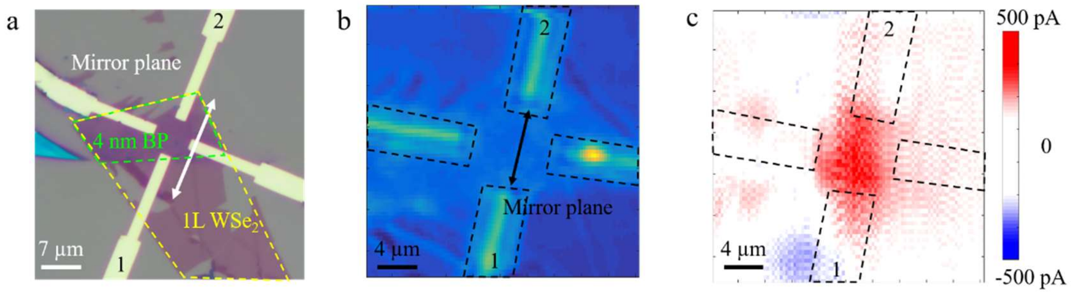

Supplementary Figure 12. BPVE generation in WSe2/4 nm BP with the 780 nm laser. **a** and **b**, Optical (**a**) and reflective (**b**) images of the WSe2/4 nm BP BPV device with two pairs of electrodes, where the electrodes E1-E2 are parallel to the mirror plane of the device. **c**, The corresponding SPCM image with the electrodes E1-E2.

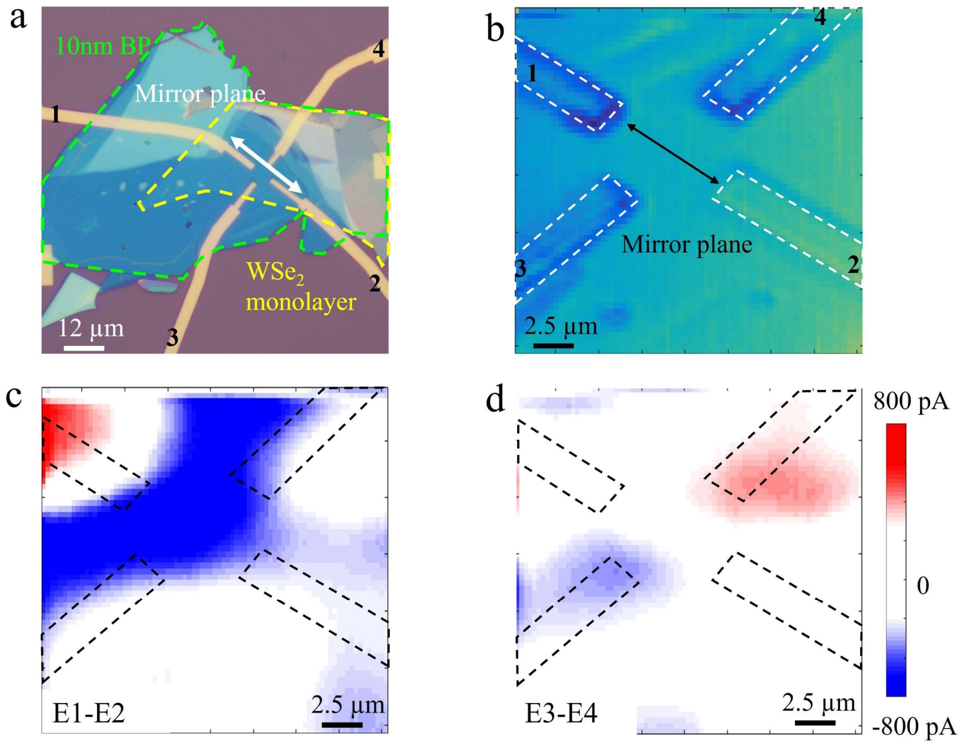

Supplementary Figure 13. BPVE generation in WSe2/10 nm BP with the 780 nm laser illumination. **a** and **b**, Optical (**a**) and reflective (**b**) images of the WSe2/10 nm BP BPV device with two pairs of electrodes, where the electrodes E1-E2 are parallel to the mirror plane of the device. **c**, The SPCM image with the electrodes E1-E2. **d**, The SPCM image with the electrodes E3-E4.

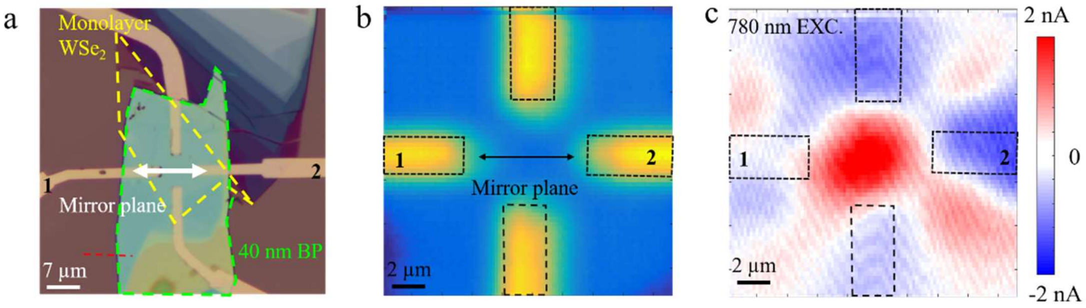

Supplementary Figure 14. BPVE generation in WSe_2/40 nm BP device with the 780 nm laser. a and **b**, Optical (**a**) and reflective (**b**) images of the WSe_2/40 nm BP BPV device with two pairs of electrodes, where the electrodes E1-E2 are parallel to the mirror plane of the device. **c**, The corresponding SPCM image with the electrodes E1-E2.

Supplementary Note 11: Supplementary results of pure BP device

The photoresponse results of pure BP device with a similar device configuration to the MoS_2/BP heterostructure were shown in Supplementary Fig. 15 and 16. Here, the electrodes E1-E2 and E3-E4 were parallel to the armchair and zigzag directions of the BP flake, respectively. In contrast to the BPVE generation in the MoS_2/BP heterostructure, when the electrodes E1-E2 were connected in the pure BP device, a typical Schottky barrier induced photovoltaic (PV) current response appeared near the electrodes while the photocurrent was negligible at the center between electrodes. In the photocurrent polarization measurement, both PV current generated from the armchair and zigzag direction displayed an anisotropic ratio similar to previous report[6]. In time-resolved photocurrent (TRPC) measurements, the BP devices demonstrate comparable intrinsic dynamics between the armchair and zigzag directions (Supplementary Fig. 15d and Supplementary Fig. 16e-h) even though there was a distinct carrier mobility difference between these two directions (Supplementary Fig. 17). These carrier mobility independent results further confirm our defect-related recombination intrinsic response model.

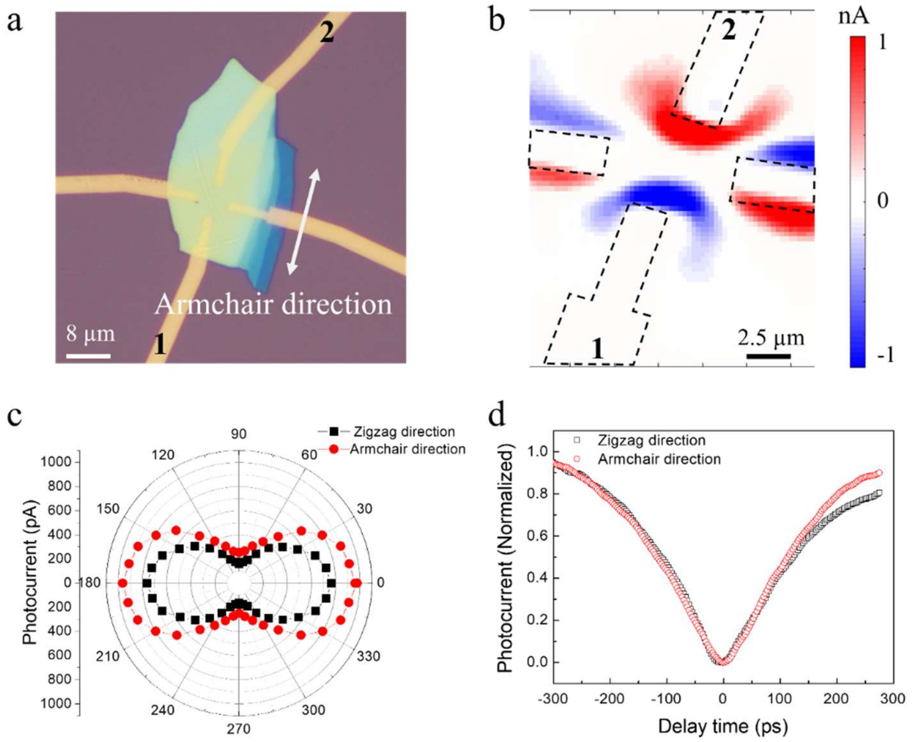

Supplementary Figure 15. Photoresponse of pure BP device with the 780 nm laser.

a, Optical image of a pure BP device with two pairs of electrodes, where the electrodes E1-E2 are parallel to the armchair direction of BP. **b**, The corresponding SPCM image with the electrodes E1-E2. **c**, Photocurrent polarization with the connected electrodes E1-E2 (red) and electrodes E3-E4 (black). **d**, TRPC results with the connected electrodes E1-E2 (red) and electrodes E3-E4 (black).

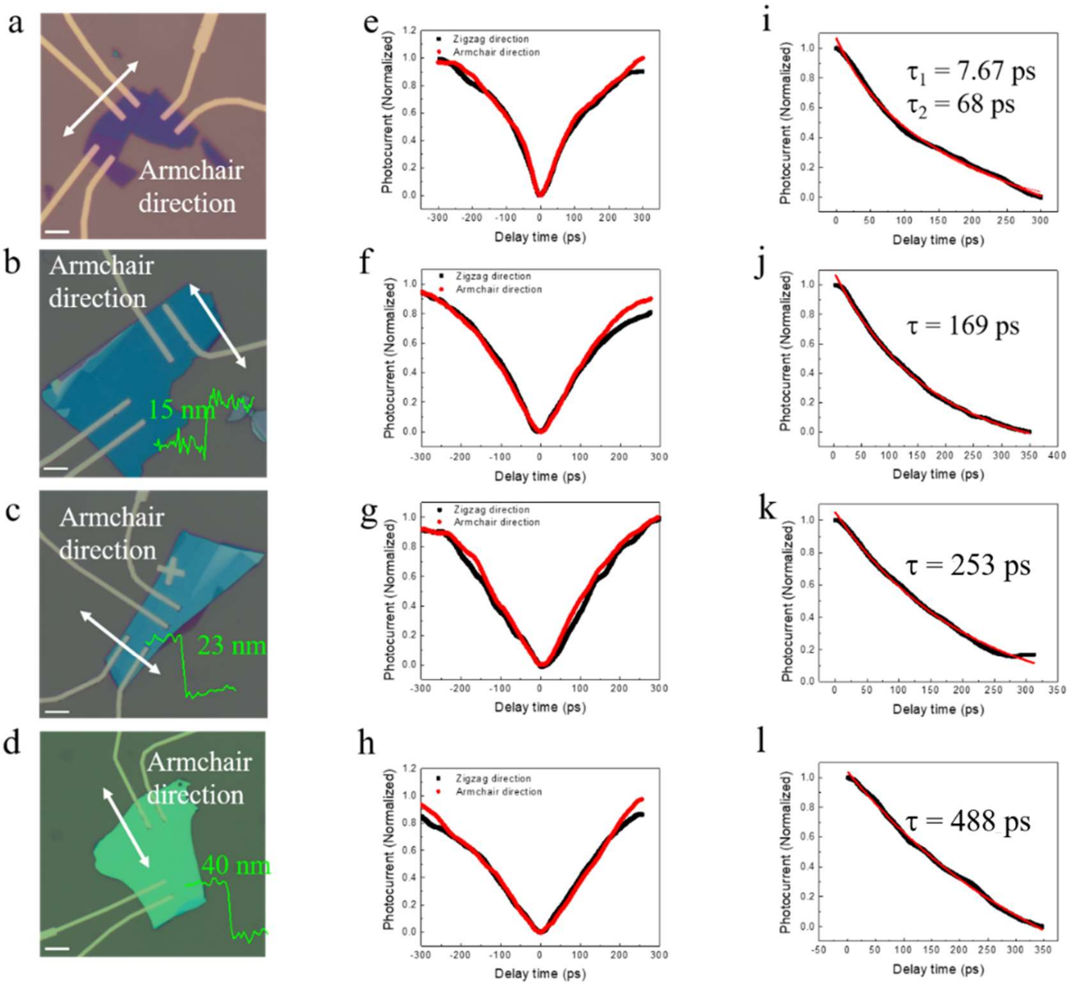

Supplementary Figure 16. TRPC results of pure BP devices with different thicknesses. a-d, Optical images of pure BP devices with different thicknesses. The white arrows indicate the armchair directions of BP. **e-h**, The corresponding TRPC curves obtained from the armchair direction (red) and zigzag direction (black). **i-l**, The corresponding normalized photocurrent (|ΔPhotocurrent|) decay curves, where the |ΔPhotocurrent| is the difference between the photocurrent (t = 0) and photocurrent (t → ∞).

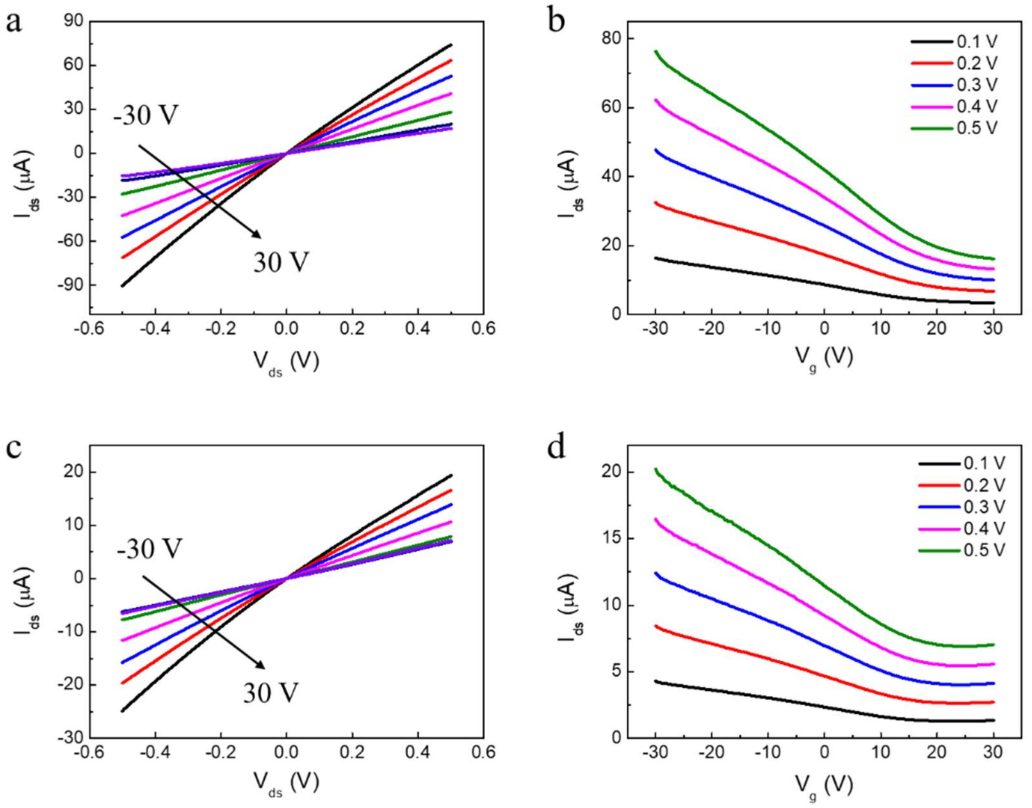

Supplementary Figure 17. Transport characterization results of pure BP device. a and **c**, Output characteristics of BP device with the electrodes parallel to the armchair (**a**) and zigzag direction (**c**). **b** and **d**, Transfer characteristics of BP device with the electrodes parallel to the armchair (**b**) and zigzag direction (**d**). The carrier mobility of BP in armchair and zigzag direction were calculated as the 256.2 $cm^2V^{-1}s^{-1}$ and 61.2 $cm^2V^{-1}s^{-1}$ using the equation: $\mu_0 = [dI_{ds}/dV_{bg}] \times [L/WC_iV_{ds}]$, where L/W is the ratio between the channel length and width, and C_i is the capacitance between the back gate per unit area (here $C_i = 1.15\times 10^{-8}$ F cm^{-2} for 300 nm thick SiO_2).

Supplementary Note 12: Band alignment simulation of MoS_2/BP heterostructure

We conducted the energy dispersion calculation in MoS_2 monolayer/BP heterostructure with aligned armchair direction by the DFT simulation and the result are shown in Supplementary Fig. 18a. Based on it we presented the band alignment of the heterostructure (Supplementary Fig. 18b), where the p-type and n-type doping for respective BP and MoS_2 monolayer together with the type-II band alignment supports our BPVE generation design.

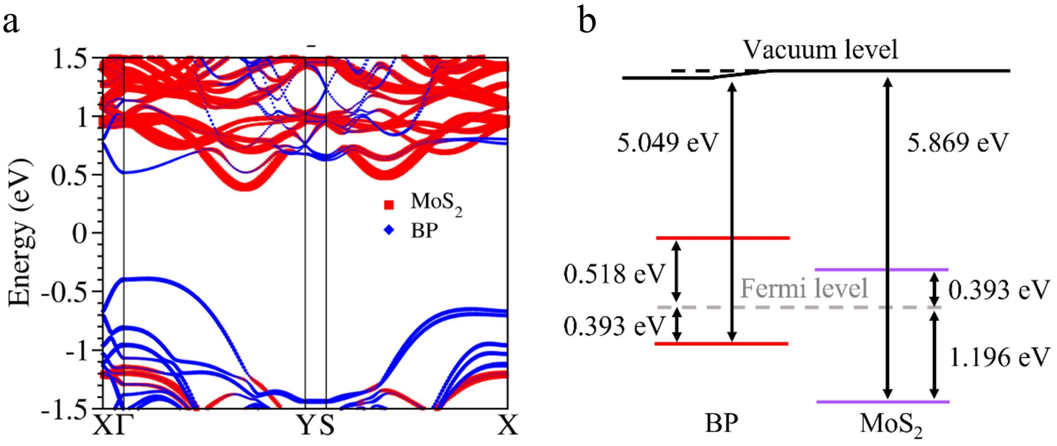

Supplementary Figure 18. Band alignment simulation of MoS_2/BP heterostructure. **a**, Energy dispersion of MoS_2/BP heterostructure. **b**, Band alignment of MoS_2/BP heterostructure.

Supplementary Note 13: Supplementary power dependent TRPC results for MoS_2/BP device

For the MoS_2/15 nm BP device shown in Fig. 3 in the main text, we conducted pump-power dependent TRPC measurements. With the increase in pump power from 108 μW to 238 μW, the generated spontaneous current demonstrates a reduced intrinsic response time from 29.29 ps to 26.65 ps, which can be explained as the enhanced exciton-exciton annihilation[7].

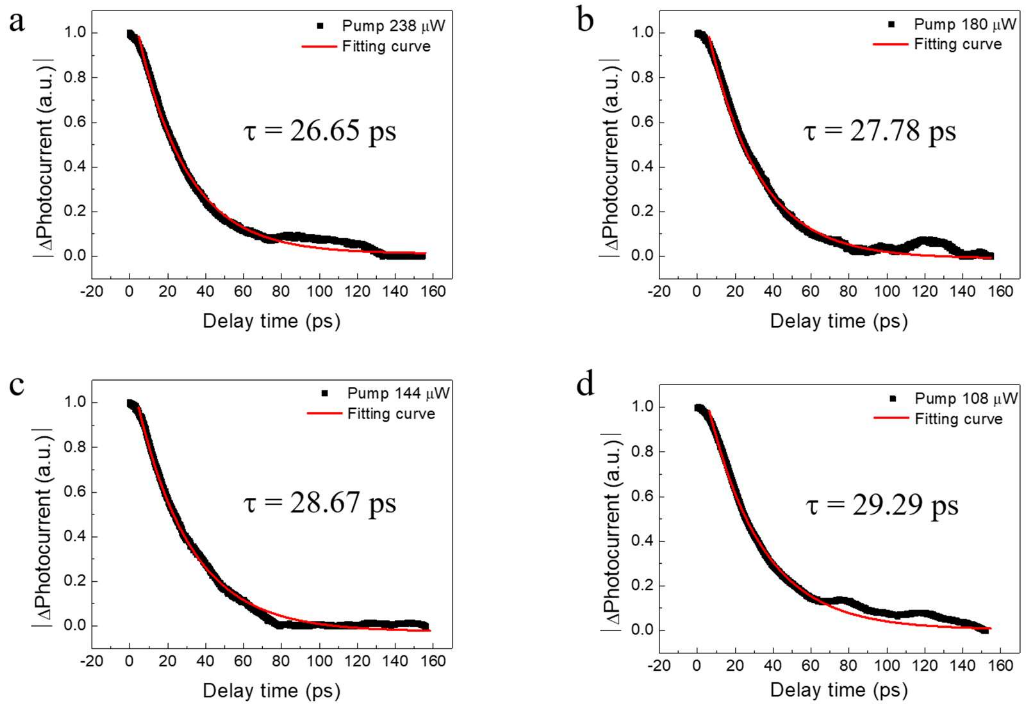

Supplementary Figure 19. Pump power-dependent TRPC measurements of MoS_2/15 nm BP device with the 780 nm laser. a-d, Normalized spontaneous current (|ΔPhotocurrent|) decay curves at the pump power from 238 μW to 108 μW, where the |ΔPhotocurrent| is the difference between the photocurrent (t = 0) and photocurrent (t → ∞).

Supplementary Note 14: Extrinsic response time of pure BP two-terminal device

Before conducting the extrinsic photoresponse in our TMD/BP BPV devices, a pure BP two-terminal device (40 nm) was first investigated. Upon the 636 nm pulse excitation, the pure BP device with 1 V external bias demonstrates a photoresponse of approximately 600 nA (Supplementary Fig. 20a), and the 90% to 10% photocurrent decay (indicated by the red dots) was calculated as 29.1 ns (Supplementary Fig. 20b).

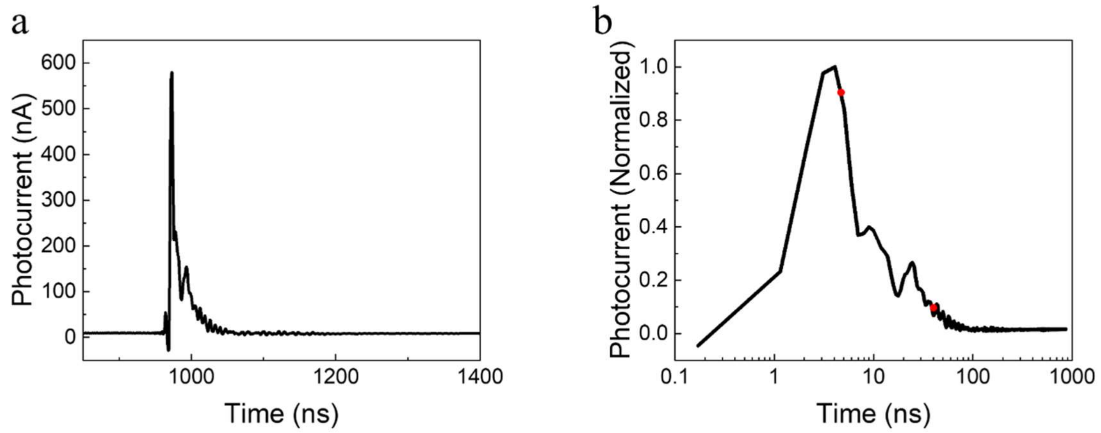

Supplementary Figure 20. Extrinsic response time of a pure BP two-terminal device. a, Impulse photoresponse of a two-terminal BP device towards a 636 nm 1MHz laser. **b**, Normalized impulse photoresponse at semi-log scale axis corresponding to (**a**). The red dots indicate 90% to 10% of the photocurrent decay.

Supplementary Note 15: Supplementary extrinsic photoresponse of MoS_2/BP device

The supplementary extrinsic photoresponse time of the MoS_2/BP device towards 779 nm illumination with different laser repetition frequencies were presented in Supplementary Fig. 21. The red dots indicate 90% to 10% of the photocurrent decay.

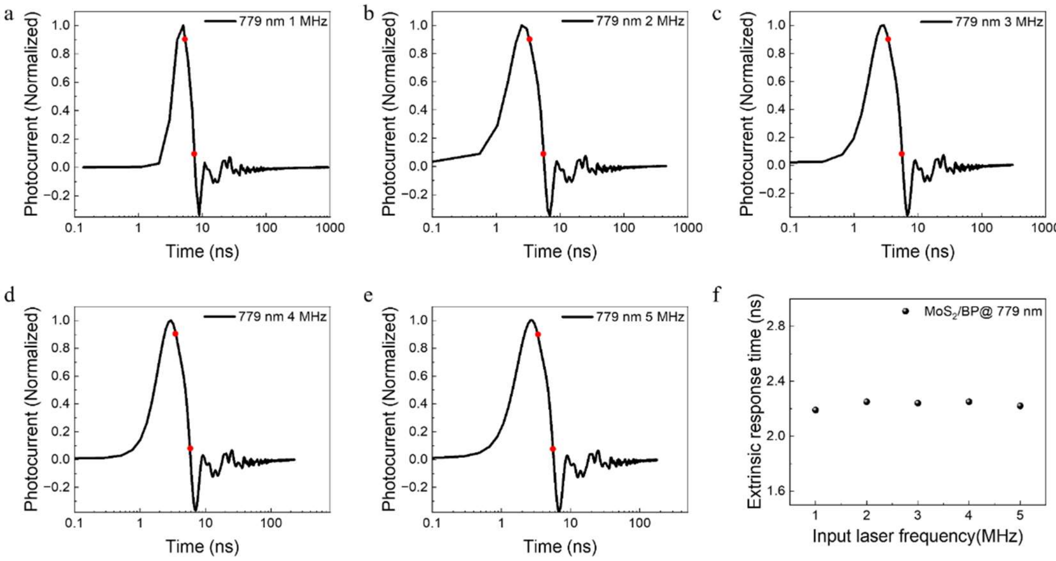

Supplementary Figure 21. Extrinsic response time of MoS_2/BP BPV device. a-e, Extrinsic response time measured in MoS_2/BP BPV device with 779 nm impulse laser at semi-log scale. The input laser repetition frequencies were changed from 1 MHz to 5 MHz as indicated in the figures. **f**, Summary of extrinsic response time in MoS_2/BP BPV device towards 779 nm laser at different repetition frequencies.

Supplementary Note 16: Extrinsic response time and 3-dB bandwidth of WSe_2/BP device

We conducted the extrinsic response time (Supplementary Fig. 22) measurements in our WSe_2/BP device under the illumination of 636 nm laser with different repetition frequencies. Here an approximately 2.2 ns extrinsic response time was obtained for different input laser frequencies. This extrinsic response time is almost equal to the intrinsic response time of approximately 1 ns measured by the TRPC technique. The 3-dB bandwidth (Supplementary Fig. 23) calculated by fast Fourier transformation was around 120 MHz.

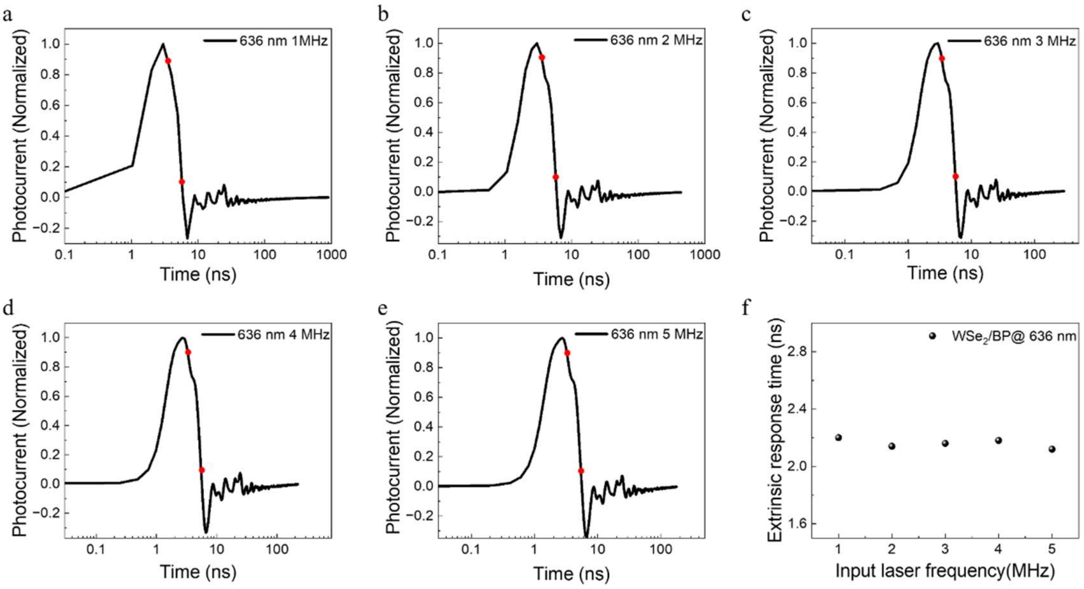

Supplementary Figure 22. Extrinsic response time of WSe_2/BP BPV device. a-e, Extrinsic response time measured in WSe_2/BP BPV device with 636 nm impulse laser at semi-log scale. The input laser repetition frequencies were changed from 1 MHz to 5 MHz as indicated in the figures. The red dots indicate the 90% to 10% photocurrent decay. **f**, Summary of extrinsic response time in WSe_2/BP BPV device towards 636 nm laser at different repetition frequencies.

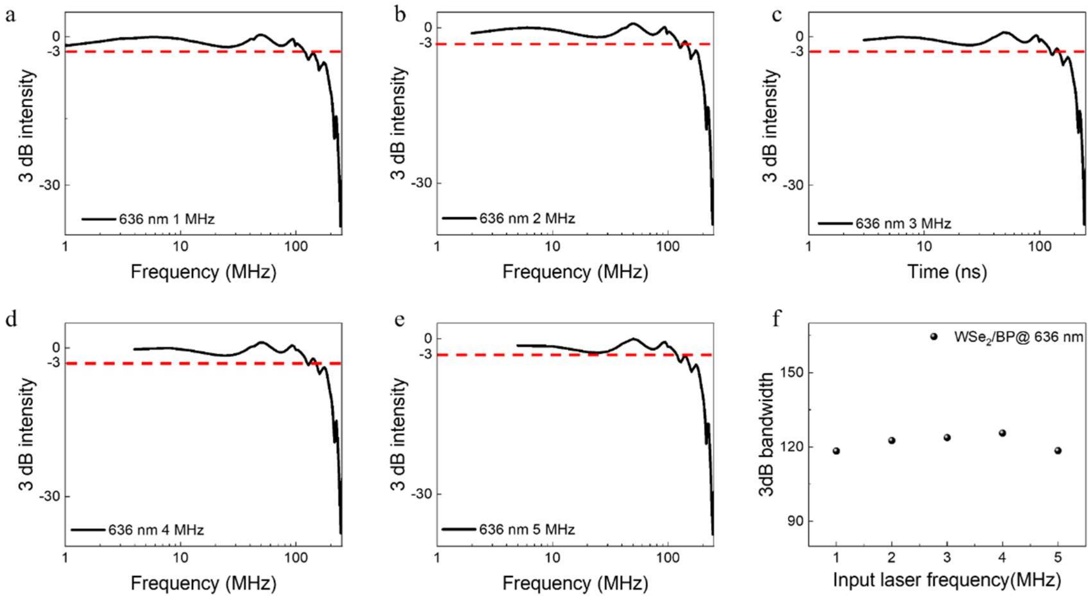

Supplementary Figure 23. Calculated 3-dB bandwidth for WSe_2/BP BPV device with 636 nm impulse laser. The input laser repetition frequencies are 1 MHz to 5 MHz for the results **a** to **e**. **f,** Summary of 3-dB bandwidth in WSe_2/BP BPV device towards 636 nm laser at different repetition frequencies.

Supplementary Note 17: Comparison of short-circuit current density in various vdW BPV devices

We compared the short-circuit current density of our MoS_2/BP and WSe_2/BP devices to the recently reported results in other vdW BPV devices with a same calculation method to that in the main text. Here, the performance of the WSe_2/BP heterostructure situated in the highest area similar to the previously reported result[12]. Meanwhile, due to the out-of-plane polarization, the BPVE strength is further enhanced in our MoS_2/BP device, indicating the improving photodetector efficiency by combining different photocurrent generation mechanisms.

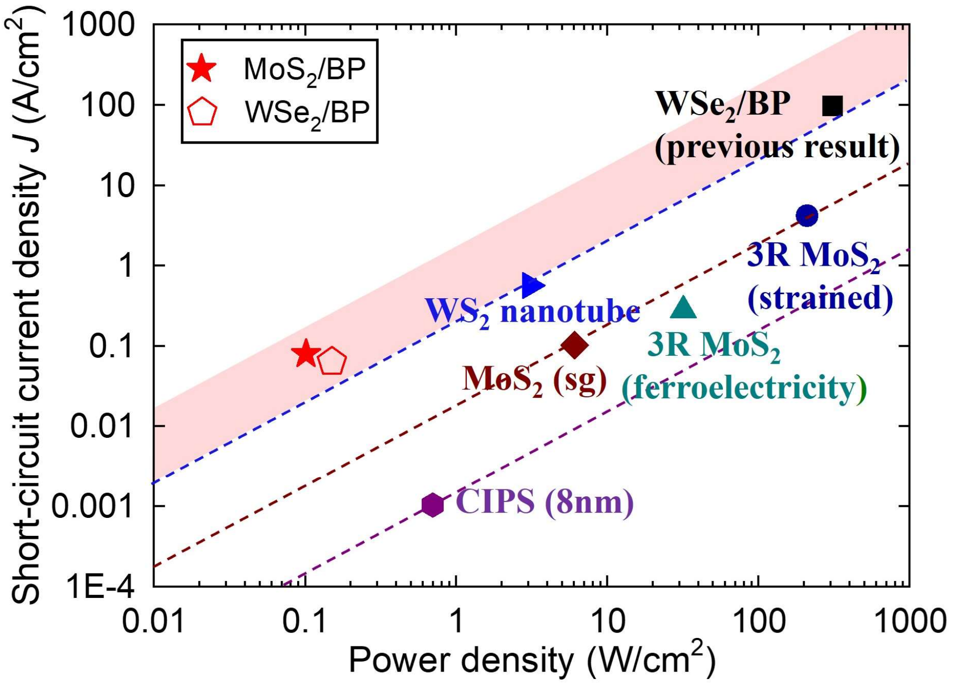

Supplementary Figure 24. Comparison of short-circuit current density in various vdW BPV devices. Data for other materials are taken from the literature (WS_2 nanotube, ref.[8]; MoS_2 (strain gradient), ref.[9]; 3R MoS_2 (ferroelectricity), ref.[2]; 3R MoS_2 (strained), ref.[10]; $CuInP_2S_6$ (CIPS) (8nm), ref.[11]; WSe_2/BP, ref.[12]).

Supplementary Note 18: Discussion of the influence of BP layers on calculations and BPVE generation

The calculations of the in-plane polarization, out-of-plane polarization, and charge carrier redistribution in the main text and above sections are based on the monolayer BP. Though these calculations are consistent with our experimental results, most of our heterostructures were fabricated with thicker BP flakes. Here, we discuss the influence of BP layers on calculations and BPVE generation. We consider two different situations. For the MoS_2/BP heterostructures with bilayer and trilayer BP, according to our calculations (Supplementary Fig. 25), there are slight potential differences from the monolayer one. Despite these small differences, the planar averaged differential charge density (Supplementary Fig. 25 b and d) is similar to the heterostructure with monolayer BP rather than the pure monolayer MoS_2, and it-induced absolute in-plane polarization strength is 0.025 eÅ for monolayer BP and 0.023 eÅ for bilayer BP, almost stating invariant. Meanwhile, the out-of-plane polarization also points from MoS_2 to BP. Hence, we consider the calculation for monolayer BP suitable for the case of MoS_2/BP heterostructures with bilayer and trilayer BP in BPVE generation.

For the MoS_2/BP heterostructures with much thicker BP, considering the generated in-plane polarization in the heterostructure is only related to the atomic layers from the hetero-interface, we argue that it would not be largely influenced by the increase in BP thickness. Besides, according to previous reports[13] with the increase in BP thickness, its Fermi level would gradually move to the valance band and the BP would become more p-doped. Thus, the out-of-plane polarization direction also does not change with the BP thickness. In summary, we are confident that our calculations based on monolayer BP would not affect the conclusions for BPVE generation in heterostructures with thicker BP.

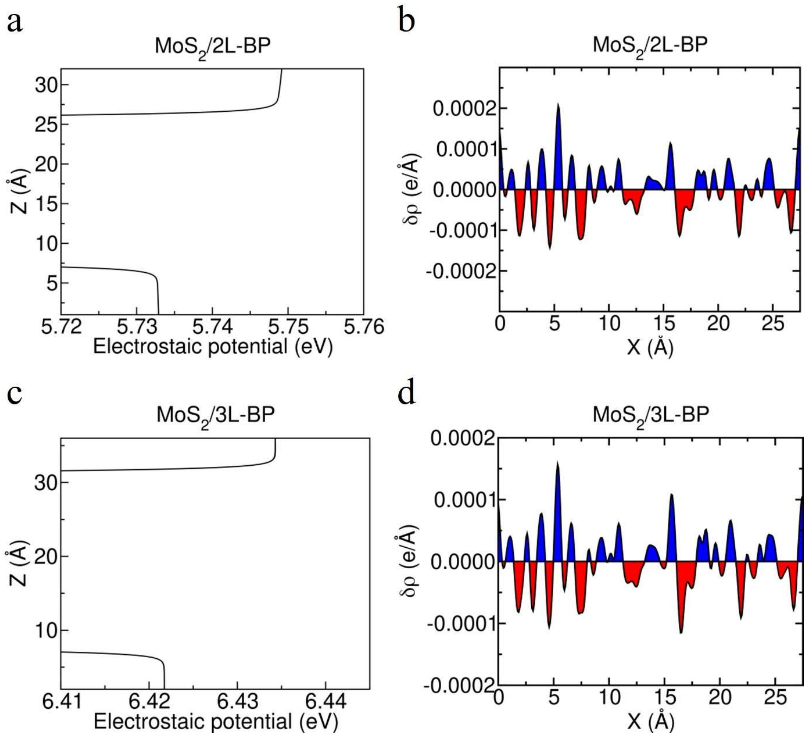

Supplementary Figure 25. DFT calculation results for MoS_2/BP heterostructures with 2L (a-b) and 3L (c-d) BP. a and **c,** Planar averaged electrostatic potential simulations along z direction. **b** and **d**, Planar averaged differential charge density.

Supplementary References:

1. Wu, J. X., Mao, N. N., Xie, L. M., Xu, H. & Zhang, J. Identifying the Crystalline Orientation of Black Phosphorus Using Angle-Resolved Polarized Raman Spectroscopy. *Angew. Chem. Int. Ed.* **54**, 2366-2369 (2015).

2. Yang, D., *et al.* Spontaneous-polarization-induced photovoltaic effect in rhombohedrally stacked MoS_2. *Nat. Photonics* **16**, 469-474 (2022).

3. Buscema, M., Barkelid, M., Zwiller, V., van der Zant, H. S. J., Steele, G. A. & Castellanos-Gomez, A. Large and Tunable Photothermoelectric Effect in Single-Layer MoS_2. *Nano Lett.* **13**, 358-363 (2013).

4. Hong, T., Chamlagain, B., Hu, S. R., Weiss, S. M., Zhou, Z. X. & Xu, Y. Q. Plasmonic Hot Electron Induced Photocurrent Response at MoS_2-Metal Junctions. *ACS Nano* **9**, 5357-5363 (2015).

5. Vanderbilt, D. & Kingsmith, R. D. ELECTRIC POLARIZATION AS A BULK QUANTITY AND ITS RELATION TO SURFACE-CHARGE. *Phys. Rev. B* **48**, 4442-4455 (1993).

6. Yuan, H. T., *et al.* Polarization-sensitive broadband photodetector using a black phosphorus vertical p-n junction. *Nat. Nanotechnol.* **10**, 707-713 (2015).

7. Yuan, L. & Huang, L. B. Exciton dynamics and annihilation in WS_2 2D semiconductors. *Nanoscale* **7**, 7402-7408 (2015).

8. Zhang, Y. J., *et al.* Enhanced intrinsic photovoltaic effect in tungsten disulfide nanotubes. *Nature* **570**, 349-353 (2019).

9. Jiang, J., *et al.* Flexo-photovoltaic effect in MoS_2. *Nat. Nanotechnol.* **16**, 894-901 (2021).

10. Dong, Y., *et al.* Giant bulk piezophotovoltaic effect in 3R-MoS_2. *Nat. Nanotechnol.* **18**, 36-41 (2023).

11. Li, Y., *et al.* Enhanced bulk photovoltaic effect in two-dimensional ferroelectric $CuInP_2S_6$. *Nat. Commun.* **12**, 5896 (2021).

12. Akamatsu, T., *et al.* A van der Waals interface that creates in-plane polarization and a spontaneous photovoltaic effect. *Science* **372**, 68-72 (2021).

13. Liu, X. C., *et al.* Modulation of Quantum Tunneling *via* a Vertical Two-Dimensional Black Phosphorus and Molybdenum Disulfide p–n Junction. *ACS Nano* **11**, 9143-9150 (2017).
